# General Index to the British and Foreign Medical Review

**Published:** 1848

**Authors:** 


Healing-process, Mr. Jones on xviii 255, 268
Mr. Travers on . . . xviii 69
nature of . . . . xviii 268
not inflammatory . . xviii 78
phenomena of. . . . xviii 76
the Art of, quotation from . x 541
Health, and beauty, connexion of . xv 53
and disease, Dr. Richter on . xv 411
and life, ancient saying on . i 368
and longevity, virtue favorable to i 361
axiom for preserving . . xvii 155
bad, a common cause of. . vii 128
prevalent cause of . . xiv 313
bill of, nature of a . . xvi 301
Board of, Mr. Chadwick on a xv 342
council of, in Paris . . v 448
new bill on . . . xix 551
difficulty of defining . . x 55
Dr. Combe on preserving xii 513, xvii 530
Dr. Hodgkin on preserving . xiii 519
economy of, by Dr. Johnson v 380, 393
preserving public . . xi 190
effects of civilization on xvii 104, xviii 237
employments on . . xx 421
human, Dr. Dunglison on . xx 519
lectures on, by Dr. Hodgkin . i 313, 362
Mr. Curtis on . . . v 380, 403
M. Royer Collard on . . xviii 154
of different classes of society . xv 336
officers of, in France . . vii 590
of mind and body, remarks on xviii 480
of the poor in Kelso . . xiii 576
Mr. Chadwick on . xv 328
of towns bill . . . . . xxi 347
Dr. Guy on . . . xxiii 240
report on . . . . xv 285
Philosophy of, by Dr. S. Smith i 313, v 380
public, duties of officers of xvii 405-6
effect of fear on . . xx 215
Mr. Martin on . . xvi 206
officers of, proposed . xvii 405
the proficient in . . v 452
Sir A. Carlisle on . . . vi 186
various treatises on . . v 380
waterproof dress dangerous to i 331
Hearder, Mr., his magneto-electric machine . . . . . xxiii 406
Hearing, on different powers of . xiv 63
Dr. Todd on . . . . viii 68, 70
organ of, Mr. Breschet on . viii 68, 69
Mr. Jones on . . . viii 68, 70
organs of . . . . . xi 222
sense of, Dr. Muller on . . viii 68, 70
the tonsils in relation to . viii 97
Heart, and arteries, functions of . xv 459
and lungs, relative position of i 428
varying velocities of xiv 182
abnormal openings in . . xx 225
aconite in diseases of . . i 249
action of, report on . . xxi 550
tincture of digitalis on . xxiii 417
adhesion of its valves, symptoms of ii 329
adhesions of the . . . xx 253
affections, Dr. Graves on . xiv 241
Heart, altered situations of . . viii 21
on alternating movements of . viii 451
alterations of, in typhus . xv 97
treatise on . . . xxii 234
an atrophied, size and weight of i 435
aneurisms of . . . vii 151, xx 224
symptoms of obscure ii 331
atrophy of, case of from Laennec ii 465
cases of . . . ii 336
causes of. . . ii 336
diagnostics of . . vi 134
its valves . . iv 142
auricular contraction of precedes ventricular . . i 436
auscultation of . . . xx 180
of the fœtal . . v 596
bellows-sound of . . . vi 133
from great loss of blood i 452
its causes . . i 450-1
without organic disease i 452
bruit de frottement of . . i 454
râlement of . . i 454
soufflet of, its causes . i 450
bruit musculaire of . . viii 449
cartilaginous changes in . . ii 326
case of wound of . . . viii 255
cases of displacement of. . viii 465
cause of dilatation of its ventricles i 439
first sound of . . viii 448
loudness in sound of . i 449
second sound of . iii 547
causes of increased size of . vi 132
motion of . . . xii 524
change of the cavities of . ii 338
seat of impulse of . xix 117
changes of, in typhoid fever . ii 50
circumscribed aneurism of . ii 460
coagula in, description of . ii 325
compared to a pump . . viii 328
concentric hypertrophy of . vii 153
congenital peculiarity of . x 587
congestion of, diagnostics of . vi 134
contraction of its orifices . ii 326
cooing and chirping sounds of i 450
coronary circulation of . . xiv 575
Corvisart on diseases of, remarks on i 425
creaking sounds of. . . i 454
curious cases of sounds of . v 168
diagnosis of diseased valves of iii 68
softening of . . . viii 464
valvular diseases of. . viii 452
digitalis in diseases of . . xx 255
dilatation of . . . . . xx 460
on the cause of . . xiv 198
of the orifices of . . ii 338
or false aneurism of . ii 338
dimensions of its different parts i 433-4
disease of, affections of brain in xx 262
and apoplexy, coincident vi 144
case of cured by veratria . ii 499
deaths from, in England ix 355
effects of phthisis on . xxiii 178
from narrowed trachea . xvi 327
from pericarditis . . xvi 328

Heart, disease of, from rheumatic diathesis xvi 468

intense headache from . ii 310

intermittent . . . ii 312

of the whole, very rare . ii 309

state of blood in . . vi 447

sympathetic effects of . ii 310

diseases of, and apoplexy, connexion of xxii 408, 420

bloodletting in . . ii 314

Bouillaud's treatment of . ii 314

chief origin of . . ii 312

classification of . i 427, ii 312

connected with Bright's disease x 316

Dr. Hope on . . viii 448, 466

Dr. Joy on . . . xii 112

Dr. Latham on xx 175, xxiii 169

Dr. Wardrop on . . v 146

etiology of . . . xx 212

from spinal irritation . i 459

from the spine . . xii 126

hereditary . . . ii 312

in British troops . . ix 70

in children . . . xvii 560

in England . . . xi 426

in old age . . . xvii 115

its orifices and valves . xx 225

M. Bouillaud on . . i 424

mercury and opium in . ii 314

miners liable to . . vi 21

modern writers on . . i 425

moral causes of . . ii 311

prevalence of . . . vi 20

prognosis in . . . ii 313

pulse deceptive in . . iii 434

their causes . . . ii 311

use of alum in . . viii 555

use of camphor in . . iii 510

with cerebro-spinal . . xxii 425

displacements of, murmurs from viii 465

Dr. Williams on first sound of viii 450

effect of some medicines on . i 264

effects of assafœtida on . . iii 510

phthisis on . . . vii 143

enlarged, bellows-sound in . i 483

expansion of its parietes . . v 420

experiments on action of . ix 561

its second sound . . i 442

false consecutive aneurisms of . ii 338

pulsation of . . . i 89

fatty degeneration of . . xxiii 177

in tuberculous cases . vi 48

more frequent in women . vi 47

female, smaller than male vi 42, vii 143

fibres of, tortuosity of . . i 429

fibrinous concretions in ii 325, viii 273, ix 386

fibrous or albuminous tissue in i 430

fluid in concretions in . . ix 388

fœtal, auscultation of . . viii 368

globular concretions in . . ix 387

hard dry sound of, cause of . i 450

Harvey's work on, remarks on i 436

healthy adult, mean weight of i 432

heat of two sides of, different xii 139

Heart, hoarse, large, rough sound of i 450

hypertrophy of, acute form of ii 335

and apoplexy . . . ii 333

curability of . xxiii 180, xxiv 563

effects of . . . vii 143

etiology of . . . xx 457

headache in . . . xxii 425

in children . . . iii 228

in the old . . . xx 254

mercury in . . . vi 387

seton in . . . . xxiv 48

signs of . . . . ii 334

three kinds of . . ii 333

treatment of ii 335, vi 387, xx 458

with dilatation . . xxiv 562

without adhesions . . xix 119

increase of, in advanced age . xxii 422

with age . . . vi 42, 45

induration of its valves, diagnosis of ii 328

in fishes, pulmonic, remark on xiv 198

inflammation of, treatment of xx 189

influenced by atmospheric changes ii 311

inorganic murmurs of . . viii 458

palpitations of . . viii 465

instance of gangrene of . . ix 434

irritability of . . . . iii 6

curious fact of . . v 114

its relation to the brain . . ii 132

to the spinal marrow ii 132, xvii 402

jarring pulse in diseases of . ix 485

largest, not most courageous . vi 42

layers of its muscular fibres . i 429

malformation of, case of . xv 154

massive concretion in . . ix 386

M. Bizot's researches on the . vi 41

M. Bouillaud's treatise on the . ii 309

measurement of vessels of . xix 117

measure of hypertrophy of . vi 44

mechanism of, Mr. Greaves on ii 599

medium of sudden affections of iii 11

metallic, ringing sound of . i 453

tinkling of . . . viii 451

minute anatomy of . . i 429

motion of, experiments on . xvii 402

motions and sounds of iii 541, xii 533

motions of, M. Bouillaud on . i 436

movements of, reflex . . xi 297

of a shark, motions of . . xiii 256

murmuring sounds of . . v 426

narrowing of its orifices, diagnosis of ii 328

of its passages, sounds indicating i 450

natural sounds of . . . viii 448

needles in parietes of . . xx 224

nervous affections of, Dr. Hope on viii 465

neuralgia of, M. Bouillaud on ii 337

new disease of, by Dr. Corrigan i 456

new displacement of . . v 425

normal dimensions of adult . xiv 239

normal situation of . . vi 132

nux vomica in diseases of . i 249

observations on diseases of . xx 453

obstructed orifices of, sounds of i 484

of the embryo, M. Baer on . i 241

Heart of an ox, weight and dimensions of i 435
bony process in . . i 435
one artery for both ventricles . iv 207
on the best preparations of . iii 506
organic unsoundness of . . xxiii 177
orifices of, widen with age . vi 44
ossification in . . . ii 326
palpitation of, musk in . . iv 399
parietal, concretions in . . ix 387
partial dilatation of . . viii 463
hypertrophy of, case of xiii 557
percussion of . . . vi 132
physiological conditions of . xx 453
plastic exudations in . . xxiv 119
polypi of, causes of . . ii 341
in pneumonia . . . xiii 386
M. Bouillaud on . . ii 340
nature and effects of . ii 341
prognosis of . . . ii 341
polypoid concretion in . . ix 386
polypus of . . . . vi 547
principle of its motion . . i 439
prolongation of its systole . i 448
proper mode of dissection of . i 436
pulse in valvular disease of . viii 456
ramollissement of . . . ii 330
dissection of . . ii 330
relation of vagi nerves to . viii 265
remarkable wound of . . x 49
remarks on wounds of . . x 51
restoration of the action of . xxi 269
rheumatic affections of vi 362, xiii 452, 456
rhythm of, its alteration in disease i 447-8
rubbing-sound of . . . i 454
rupture of, Mr. Thurnam on . v 607
sanguineous concretions of . ix 549
saw-sound of . . . . i 450
scraping sound of . . . i 454
secondary disease from that of the ii 310
sibilant sound of, causes of . i 452
signs of adipose disease of . viii 464
size of forming hypertrophy . i 432-3
size of its cavities unequal . i 432
smaller in tubercular subjects vi 43
softening of . . . . xxiii 181
sound of muscular extension of viii 449
sounds and actions of . . ii 598
sounds of v 589, xix 105, xx 177, 454
and their causes . . i 440
Dr. Hope's experiments on i 445, 446
Dr. Williams on . . i 445, 446
experiments on . . i 445
from friction . . . i 454
in diseases of . . . xiv 178
in pericarditis . i 454, xxiv 551
known to Harvey . . i 436
M. Piorry on . . . vi 133
M. Rouanet's theory of . i 445
right and left sides of distinct i 484
summary of hypotheses on i 444
their audible extent . i 440-1
valvular theory of . i 445
variation in intensity of . i 449
Heart, sounds of, various theories of i 441-3
spasm of, remarks on . . ii 338
treatment of . . xxiii 184
state of, in typhus fever . viii 281
stroke of, against the side . i 437
canvassed . . . i 438
experiments on . i 437-8, xii 526
structure and functions of . x 238
of its auricles . . . i 430
of its ventricles . . i 429
suppuration of . . . ii 330
symptoms of polypi of . . ii 341
the pulse in diseases of . . ix 485
tricuspid valve of, Mr. King on ii 216
triple and quadruple sounds of i 452
tubercles of . . . . xx 225
tubercles in, case of . . xvi 385
ulceration and perforation of . ii 331
unsound, secondary diseases from xxiii 182
on dropsies from . . xxiii 183
use of ganglionic system to . iii 11
valves of, their functions . iv 364
valvular click of . . . viii 449
disease of . . ii 324, xx 254
mixture for . . ii 229
signs of . . xiv 245
murmurs of . . . viii 453
various positions of . . xix 104
vegetations or granulations in . ii 325
ventricles of, can hold a hen's egg i 434
capacity of . . . vi 43
thickness of . . . vi 43
volume of, no single standard for vi 42
weight and dimensions of . i 433
weight of . . . . . vii 143
increases with age . . vii 143
white spots on . . . xi 448
nature of . . iv 43
why liable to rheumatism . i 431
wounds of, cases of recovery in vi 171
not always fatal . . vi 170
Hearts, diversity of individual . ix 487
hairy, stories of explained . iv 42
hypertrophied, sizes and weights of i 435
impulse in pericarditis . . xxiv 552
lymphatic, of tortoises . . x 256
Volkmann on . . . xxi 565
two, in one pericardium . . xiv 255
Heat, anatomy and physiology of . xix 260
and cold, in relation to the nerves iii 15
and respiration, Dr. Thomson on xiv 297
animal, Chossat's experiments on xvii 431
disquisition on . . xi 393
development of . . xiii 484
Dr. Billing's theory of . v 202
Dr. Davy's experiments on xii 139
Dr. Metcalfe on . . xviii 520
Dr. Paine's doctrine of . xi 394
experiments on . . xiii 229
first truly explained . i 383
John Hunter on . . v 50
Liebig on . . . xiv 298
Mr. Jeffreys on . . xvii 153

Heat, animal, Mr. Paget's report on . xxi 557

not upheld by alcohol . xxiv 526

oxidation the cause of . xiv 301

present knowledge of . xix 266

production of . . . viii 271

source of . . . xiv 298

theory of, authors on . xiv 297

as a cause of disease . . xiii 348

as a counter-irritant . . vii 62

as a remedial agent, M. Guyot on ii 266

central, Mr. Taylor on . . xxii 229

Dr. Crawford's theory on, opposed xvi 195

Dr. Murray on the influence of xiii 349

employment of, in ulcers, &c. xix 182

evolution of, by plants . . ix 545

facts relative to, from Hunter . vii 524

generation of, at death . . xvii 431

hastens putrefaction in water . ii 400

hydropathic abstraction of . xxii 444

in incubation of reptiles . xii 529

in relation to nervous system . xii 149

to respiration . . . xii 148

to state of blood . . xii 147

in ulcer, its effects tested . ii 265

its effect on muscular energy . xiv 503

Liebig's theory on, opposed . xvi 195

M. Guyot's vital theory of . xix 182

nervous system in relation to . xi 395

of trees, experiments on . . xi 396

of two sides of heart, different xii 139

pungent, a sign of pneumonia . ix 489

respiration in relation to . xi 394

'Heat' or 'rut' in animals . . xvii 271

Dr. M. Edwards on i 333

vagina black in . xiv 210

Heated air, effects of on wounds . ii 264

Hebdomadal or heptal cycle . . xviii 166

periods, Dr. Laycock on . xii 64

Hebra, Dr., his history of surgical operations . . . . . xvi 46

Hecker, Dr., on epidemics of the middle ages . . . xvii 522

on gangrene from obstruction . xiv 84

medicine in Prussia . . i 289

medical societies in Prussia ii 577

the plague . . . v 191

Hectic, a cause of visceral disease . ii 12

in relation to operations . ii 12

syphilitic, treatment of . . v 24

night-sweats, treatment of . xix 23

Hectic fever, a healing process . xv 411

crookedness of nails in . xii 95

distilled vinegar in . . xix 23

Dr. Clutterbuck on . v 188

Mr. Morgan on . . x 535

nature of . . . xii 95

senile, remedies in . . xvii 108

small bleedings in . . v 188

use of digitalis in . . v 189

sweat said to be purulent . i 509

Hedenus, Dr., obituary of . . vii 298

Hedgehog, bat, and dormouse, heat in xii 148

Heidelberg, extract of a letter from iv 282

Heidler, Dr., on blood and nerves xxii 218

on the cure of gout . . xiv 334-5

on the Marienbad waters xiv 309, 319

Height of body, morning and evening v 136

Heights, effects of air at great . xvii 277

Heim, Dr., his medical works . iv 492

his mixture for chronic peritonitis xxiv 47

on vaccination . . vii 186, 210

Heine, Dr., his new bed for fractures, &c. xvi 486

on spontaneous dislocations . xvi 486

Heister, his allusion to constitutional irritation . . . ii 1

Helicine arteries of Müller . . xiv 295

Helicotrema in the cochlea . . viii 79

Heliodorus, his papyrus bougies . xiii 316

Hellebore, its use in quartan ague . ii 177

Helm, Dr., on puerperal diseases . xiii 100

Helminthiasis as contagion . . xx 406

Hemal arches in the heads of fish . xxiii 487

Hematemesis, Dr. Stokes on . . ix 476

on emetics in . . . xxi 368

fatal case of . . . . xx 239

with chlorosis . . . xix 345

Hematite, as an antidote for arsenic iv 239

Hematocele mistaken for fungous testicle i 476

Hematomania by Schneider . . vi 515

Hematophobia, German, exposition of xvi 475

Hematosine, properties of . . vi 437

proportion of, in blood . . vi 439

same in man and animals . vi 437

Hematuria, causes of . . . xv 480

duration of . . . . xv 479

endemic, in the Mauritius . xv 481

from inflamed kidney . . viii 150

idiopathic . . . . xv 481

observations on . . . xv 478

Hemeralopia, epidemic . . . xii 538

Hemicrania, and neuralgia, cure of x 262

nature and treatment of . . xvii 418

treatment of . . . . . xxiv 41

Hemiplegia, connected with syphilis xx 259

convulsion of arm in . . v 503

effects of emotion in . . xi 457

experiment by Nature in . xix 305

facial, in newborn infant . xx 552

of infants . . . x 269

from obstructed circulation . xx 259

interesting case of . . . x 585

observations on . . . xx 35

proportional danger in . . xiv 56

seat of emotion in . . . xi 457

Hemispherical ganglia, Mr. Solly on xxiv 573

Hemite or hemitis, on the term . vi 136

Hemlock, in cancer, Lisfranc's use of xiv 15

Hemlock-baths in diseases of the skin vi 538

-water-dropwort, poisoning by xviii 563

Hemodynamometer, M. Poiseuille's viii 336

Hemoptysis, Dr. Chapman on . xxi 366

from pulmonary artery . . xvi 248

from the bronchial artery . xvi 248

in children . . . . xx 99

in phthisis, M. Louis on . xvi 440

remarks on . xviii 159, xix 116

Hemoptysis, in relation to phthisis . ix 325

ipecacuanha emetics in . . xxi 366

on spots in the lungs in . . xi 409

pulmonary apoplexy . . xvi 248

treatment of . . . xvi 249, xxi 107

Hemorrhage, after delivery . . xvi 320

after ligature, causes of . . xxii 100

after lithotomy . . . . xiv 234

after placenta, in 16,434 labours ii 72

arresting . . . . . viii 327

by perplication . . viii 327

bloodletting in . . . . x 64

caused by emotion . . . . x 62

causes of . . . . . x 60-4

cerebral, coma in . . . xxii 405

in children . . . xvii 301

in newborn infants . . vii 92

its relation to palsy . i 142

observations on . . xx 35

colourless . . . . . xxiv 138

compression in . . . . xii 442

death from sudden . . . xviii 220

deaths from, in England . ix 356

disposition to, in families . x 63

Dr. Carswell on . . . i 141

Dr. Chapman on . . . xxi 366

during labour, neglect in . ii 81

effect of temperature on . x 61

elementary conditions of . x 59

etiology of . . . . . x 60

expectant treatment of . . x 64

from bowels remark on . . vi 139

erectile tumour of uterus ii 571

internal perforation . . xxii 372

meteorological changes . x 61

the ureters . . . xv 479

the urethra . . . xv 479

uterine polypi . . xiii 543

polypus . . . xvii 509

varicose veins . . i 143

hazard of styptics in . . x 66

in early gestation . . . xii 118

influence of age on . . x 62

into brain in children . . xvii 553

into pericardium, case of . v 607

meteorology of . . . xx 212

M. Andral on the blood in . xvii 149

M. Gendrin on . . . x 59

Mr. Guthrie's precept on . vi 452

Mr. Mayo on arresting . . iii 69

on death by . . . . v 175

dilating os uteri in . . iii 414

internal medicines for . x 66

slight pressure in . . xi 309

palmar, case of . . . v 463

on arresting . . . v 463-4

placental, *not* slow . . ii 81

post-partum, ergot of rye in . xxiii 289

theory on . . . xxiii 284

promoted by sleep . . . x 63

pulmonary, use of cold in . ii 532

renal, idiopathic . . . xv 481

M. Rayer on . . . xv 478

Hemorrhage, renal, singular case of . xv 479

restraint of, in insects . . xx 506

rupturing membranes in . xiv 368

Sir C. Bell on . . . vi 157

secondary . . . . xii 450

in aneurism . . . xxiv 419

sub- and intra-arachnoid . xxi 410-11

surgical treatment of . . xii 441

temperament favorable to . x 62

theory of . . . . iii 211

treatment of . . . . x 64

uterine . . . . . xx 458

caution on cold in . . xv 528

cold injections in vagina in ii 85

shock in . . . ii 85

diagnosis in . . . xiv 371

Dr. Collins on . . ii 82

Dr. Davis on . . . iii 408

Dr. Gendrin on . . x 77

ergot of rye in . . viii 250

friction in . . . ii 85

from tumours . . vii 450

new method in . . xii 261

opium in . . . xxi 107

pressing aorta in . . xix 420

plug in . xiv 368, 371, xv 528

report on . xvii 539, xxiii 284

rupturing membranes in . iv 406

sponge as a plug in . vi 85

transfusion in . . . xi 260

treatment of iii 409, v 578, viii 250, xiv 368, xv 528

valuable fact on . . ii 83

value of plug in . . ii 82

with exhaustion . . . iii 212

irritation . . . iii 211

placenta normal . . x 81

women more subject to . . x 62

Hemorrhages, critical . . . xxi 23

prevalent in winter . . xxiv 103

traumatic, Dr. Beck on . . iv 154

Hemorrhagia cerebri, &c., case of . xxii 387

Hemorrhagic diathesis, fatal case of xiv 578

treatment of . xiv 577

tendency in a family . . ix 247

Hemorrhoidal blood, analysis of . xvi 227

erectile tumours, case of . xxii 474

habit, remarks on . . . xx 314

tumours, Dr. Bushe on . . iv 470

ointment for . . . i 579

Hemorrhoids, different species of . v 482

dyspepsia from . . . xix 28

from dilated veins . . . v 482

inflammation in . . . v 483

in submucous tissue . . v 482

internal, on ligature in . . v 484

M. Lisfranc on . . . xviii 34

Mr. Mayo on . . . iii 76

nitrate of silver in . . . xiii 62

of erectile structure . . v 483

pain of toes, &c., from . . iv 133

treatment of . . . xviii 34, xxi 368

Hemorrhois, a cause of bleeding in xii 116

Hemorrhois bleeding, arterial in . xii 117
Hemotrophy different from hyperemia xi 346
Hemp, common, intoxicating properties of . . . . . xxiii 219
euphorbia peplus, noxious to . iv 130
Indian, doubtful efficacy of . xvi 357
experiments with . . x 226
inebriating drink from . x 225
medical effects of . . xviii 368
or Gunjah . . . x 225
preparations of . . x 226
use and effects of . xxiii 217-20
use of, in diseases . . x 226
narcotic effects of . . . x 225
Hemp-water, not insalubrious . v 451
Henbane and emetic tartar, Dr. Philip on ii 146
HENDERSON, Dr. A., on sea scurvy viii 545
HENDERSON, Dr. W., on fever in Scotland, xviii 179, 181
on homœopathy . . xxi 225, 249
typhus fever . . xii 293, 313
HENFREY, Mr., his Outlines of Botany xxiv 223
HENKE, Dr., journal of . . . . iii 196
HENLE and KÖLLIKER on the Pacinian corpuscles . . . . . xix 78
HENLE, Dr., his General Anatomy . xiv 478-9
his investigations . . . ix 398
his Rational Pathology . . xxiv 289
on contractility of vessels . x 551
on the cuticle . . . vi 333
strictures on his theories . ix 410
HENOCH, Dr., his clinical observations xxiv 41
HENRY, Dr., obituary of . . ii 612
HENRY, Dr. J., on bilious complaints vii 476
HENRY, Dr. W., on the laws of contagion i 369
HENSLOW, Mr., his Principles of Botany iv 1, 3
Hepatic abscess, absence of pain in iii 341
absence of pyrexia in . iii 341
central . . . . . iii 341
Dr. Murray on exploring xiii 359
fatal cases of . . . xxiii 120
gall-bladder mistaken for ix 482
incipient stage of . . iii 340
in dysentery . . . xxi 45
in India, Mr. Geddes on . iv 279
Mr. Geddes on . . iii 340
new mode of opening . ix 483
on opening . . . xxi 48
on puncturing . . . xii 89, 90
on the outlet of . . xxi 47
purulent metastasis in . xii 91
rules for exploring . . xiii 360
secondary cause of . . xxiii 150
symptoms of . . . iii 340
treatment of . . . iii 342
with dysentery . iii 341, xxiii 149
abscesses, exploration of . . xv 246
affections, mercury in . xii 87, xxi 48
mineral waters in . . xii 85
asthenia, treatment of . . xii 86
disease, pain of shoulder in . vi 140
relative frequency of . xiii 360
inflammation, in India . . xxiii 120
Hepatic inflammation, treatment of xxiii 125
irritation, M. Bonnet on . xii 78
treatment of . . xii 84
region, abscess over the . . ix 483
Hepatitis, acute, symptoms of . iii 337
with inflammation of Glisson's capsule . iii 337
a disease mistaken for . . ix 483
causes of, Mr. Twining on . iii 338
chronic, on purgatives in . xii 84
congestive, symptoms of . iii 337
consecutive, causes of . . xii 81
cupping and blisters in . . xii 85
deep-seated, symptoms of . xiii 358
different forms of . . . iii 337
evacuations of pus in . . vi 251
in India, causes of . . . xxiii 152
use of mercury in . . xii 91
in relation to dysentery . . iii 334
to peritonitis . . . xii 83
leeches to anus in . . . xii 85
mercurial inunction in . . viii 420
mercury in . . . . . xix 31
Mr. Conwell on causes of . iii 338
nitro-muriatic bath in . . xii 85
primitive causes of . . . xii 80
tension of rectus abdominis in xiii 359
treatment of . . . iii 339
urea in the urine in . . xxiii 153
Hepatization, hemorrhagic, of lungs iii 259
of the lungs, on gray . . xi 407
on red . . . . . xi 406
Hepatized lung, remarks on signs of iv 315
Hepato-pleuro-bronchial fistula . xiii 236
Herb-doctors of Ireland . . xviii 37
Herbivorous animals cannot vomit ii 538
nutrition in . . xiv 507, 515
Hereditability of disease . . xxiv 303
Hereditariness, Dr. Levy on . . xix 55
of insanity . . . . xiii 259
Hereditary cerebral affections . xx 33
disease, Dr. Holland on . . viii 484
remark on . . . vi 20
diseases, remarks on . . xxii 142
treatise on . . . xvii 247
predisposition to ague . . xx 211
remarks on . . . xx 315
HERING, Prof., on cowpox in the cow ix 76, 78
Hermaphrodism, classes of . . viii 26
Dr. Knox on . . . viii 27
feminine . . . . viii 28
masculine . . . . viii 27
mixed . . . . . . viii 28
neuter . . . . . . viii 28
production of . . . viii 26
singular case of . . . xxi 469
St. Hilaire on . . . viii 25
with excess of parts . . viii 29
Hermaphrodite, case of a supposed xii 332
Heroic remedies, remarks on . . xxii 237
treatment, mischief of . . v 567
HERON, Dr., on the treatment of ozæna i 270
HEROPHILUS, account of his works xv 109

| | | |
|---|---|---|
| HEROPHILUS, amusing anecdote of | xv | 108 |
| birthplace and time of | xv | 108 |
| Dr. Marx's account of | xv | 106, 112 |
| his dissecting criminals alive | xv | 109 |
| obstetric practice | xv | 113 |
| surgical practice | xv | 113 |
| medical opinions of | xv | 110 |
| pharmaceutics of | xv | 113 |
| Herpes, and its species | xv | 388 |
| circinnate group of | xv | 388 |
| new mode of treating | iii | 241 |
| phlyctenoid group of | xv | 388 |
| remarks on | ii | 149 |
| urodialytic of old age | xvii | 117 |
| Herpes circinnatus, iodine in | viii | 591 |
| Herpes zoster, structure of the vesicles in | ii | 149 |
| HERSCHEL, Sir J., on the eye | xii | 217 |
| HERZOG, Dr., on insanity | xxi | 460 |
| Hernial sac, blow on, treatment of | xvi | 319 |
| contraction of neck of | xxii | 202 |
| hydrocele of the | xvii | 73 |
| Mr. Teale on the | xxii | 194 |
| rare contents of an | iii | 562 |
| Hernial trusses, M. Malgaigne on | xiv | 104 |
| want of in Turkey | iv | 396 |
| Hernia cerebri, case of | ix | 221 |
| Mr. Guthrie on | xv | 178 |
| treatment of | ix | 221 |
| Hernia, on acetate of lead in | vi | 537 |
| age in relation to | xiv | 93 |
| and varicocele, diagnosis of | xiv | 107 |
| an insidious inflammation in | xv | 40 |
| belladonna in | ii | 263 |
| block of Dr. Hood for, *figure of* | iii | 235 |
| Mr. Chase for, *figure of* | iii | 235 |
| calomel and opium in | xiv | 110 |
| in small doses in | iii | 361 |
| case of ruptured strangulated | xi | 448 |
| cases of, by Mr. B. Cooper | ix | 392 |
| of, unfit for taxis | xv | 40 |
| cause of strangulation in | xxii 51, iii | 76 |
| causes of | xiv | 94-6 |
| and effects of | xxii | 196 |
| cold douche in | xiv | 109 |
| congenital | xiv | 98 |
| crural, in women, causes of | xiv | 107 |
| frequency of | xiv | 105 |
| cured on Dr. O'Beirne's plan | vii | 584 |
| cure of by trusses | vi | 343 |
| in children | viii | 562 |
| danger of tobacco enemata in | xv | 41 |
| diagnostics of stricture of | xiv | 100 |
| diaphragmatic, fatal case of | iv | 479 |
| division of stricture in | xxii | 377 |
| entero-vaginal | iii | 396 |
| evaporation of ether on | xv | 42 |
| external division of structure in | xxii | 200 |
| femoral, complications of | xxii | 206 |
| seat of stricture in | xxii | 207 |
| general mortality in | xiv | 96 |
| golden incision for | vi | 345 |
| humoralis, compression in | ii | 253 |
| from gonorrhœa | xii | 371 |
| Hernia humoralis, use of iodine in | viii | 523 |
| improved knowledge of | x | 102 |
| incarcerated, suction-pump in | i | 586 |
| incomplete inguinal | iii | 237 |
| in different countries | xiv | 95 |
| inefficient treatment of | viii | 561 |
| inguinal, anatomy of | xii | 511 |
| and crural, diagnosis of | xiv | 106 |
| hereditariness of | xiv | 97 |
| immediate causes of | xiv | 98 |
| in women, causes of | xiv | 106 |
| frequency of | xiv | 105 |
| operation for | xxii | 205 |
| radical cure of | xiv 101, xiv | 232 |
| reduction of | x | 268 |
| seat of stricture in | xiv 99, | 100 |
| various aspects of | xiv | 98 |
| inguino-interstitial | iii | 237 |
| in the young, on trusses to | xiv | 99 |
| Turkey, an operation for | iv | 396 |
| royal suture for | vi | 345 |
| irreducible femoral, pad for | xxii | 208 |
| treatment of | xxii | 199 |
| intra inguinal | iii | 237 |
| intra-parietal, after wound | xiv | 556 |
| interstitial | viii | 562 |
| or incomplete | xxii | 203 |
| large, M. Gerdy on taxis in | ii | 566 |
| Lisfranc on operating for | xv | 38 |
| on reduction of | xv | 38 |
| Litrica, diagnosis of | xiv | 363 |
| Dr. Riecke's cases of | xiv | 365 |
| history and cause of | xiv | 361 |
| nature of | xiv | 360 |
| prognosis of | xiv | 363 |
| progress of | xiv | 361 |
| strangulation of | xiv | 362 |
| treatment of | xiv | 364 |
| local bloodletting in | xv | 40 |
| long-continued taxis in | xv 39, | 41 |
| mercurial ointment in | xv | 41 |
| mischief from bandages for | viii 562, | 563 |
| mode of curing by M. Belmas | vi | 346 |
| modifications in operation for | ii | 566 |
| mortality from operations for | xv | 38 |
| moveable, treatment of | xxiii | 34 |
| M. Bonnet's method of curing | vi | 345 |
| M. Gerdy on the treatment of | ii | 566 |
| M. Gerdy's mode of curing | vi | 348 |
| M. Malgaigne on | viii | 561 |
| Mr. Liston on operating for | xxii | 378 |
| Mr. M. W. Hilles on | vi | 203 |
| Mr. Teale's treatise on | xxii | 193 |
| nature and formation of | vi | 342 |
| neglect of commencing | viii | 562 |
| new instrument for | ii | 566 |
| species of strangulated | x | 565 |
| variety of | xii | 546 |
| oblique inguinal | viii | 561 |
| obliteration of opening in | vi | 344 |
| obstacles to taxis in | vi | 559 |
| occupations in relation to | xiv | 95 |
| of appendix cæci vermiformis | i | 583 |

Hernia of diaphragm . . . xxiv 178

diverticulum of intestine . xiv 360

Fallopian tube, case of . x 267

testes . . . . xvii 80

thyroid foramen . . xiv 556

tunica vaginalis . . . xxii 204

uterus, case of . . . xii 261

omental sacs in . . . xx 93

on cold, for reduction of . xv 42

delay in operating for . xv 38

opening the sac in . iii 358, vi 68

opium before operating for . iii 358

some states of the sac in . iii 362

operation for, in 107th year of age xvii 276

for radical cure of . xii 332

for, sac not divided . iv 256

operations for, history of . xvi 57

pads for, on the form of . xiv 102

progress of . . . . . viii 562

purgatives after reduction of . xv 41

radical cure of . . vi 341, xi 252

by injection . . . xx 56

rest . . . . . x 569

truss . . . . v 300

of in America . . . iii 234

of, remarks on . . vi 351

cures of, by trusses . . xiv 104

reducible, neglect of . . viii 561

radical cure of . xiv 232, 234

cures for . . xxii 198

reducing, surgeon's position in xv 42

report of cases of . . . xviii 336

sex in relation to . . . xiv 92

statistics of . . . . . xiv 91

stramonium poultice in . . iii 359

strangulated, after-treatment of xiv 110

age in relation to . . xxii 197

cured by a quack . . iii 359

curious case of . . vi 391

danger of puncturing in . ii 201

Dr. Parrish on . . iii 355

injections in . . . iii 360

Malgaigne on . . . xiii 245

Mr. King on . . . vii 456

mesenteric . . . vii 290

morphia in . . . xiv 581

new treatment of . . vi 558

on open bowels in . . xxii 198

opium in . . . xiii 566

purgatives in . . . xiv 109

reduced "en masse" . xviii 367

the taxis in . . . vi 559

tobacco enema in . . xiv 109

treatment of . . . xiv 109

tube for . . . . vi 559

strangulation in, Malgaigne on xiv 100

subcutaneous operation for . xii 556

taxis in, direction of the . . xv 40-1

observations on . . xxii 199

relaxing muscles in . xv 41

the abdomen in relation to . xiv 97

treated on Dr. O'Beirne's plan . viii 596

treatment of . . . . xxii 52

Hernia, treatment of, in Turkey . iv 396

omentum in . iii 362

triangular pad for . . . xiv 103

trusses for . . . . . xiv 101

two cases of . . . . vi 68

principles of cure of . . vi 342

sacs in some cases of . ix 393

tying neck of sac in . . vi 345

umbilical, M. Malgaigne on . xiv 108

use of scissors in . . . ii 566

varicose tumour, simulating . xiv 235

varieties of stricture in . . xv 42

various constituents of . . xxii 195

treatises on . . xiv 91

ventral, case of . . . xii 433

winged-director for . . xxii 201

without peritoneal sac . . xii 546

with sphacelation, case of . vi 70

Hernia pericardii, case of . . iv 532

Hesse, endemic bronchocele in . xx 170

topography and statistics of . xx 171

Hessler, Dr., his edition of Susruta xxiii 521

Heteradelph monsters . . . xii 375

Hetero-plastic formations . . xiii 339

Heterotaxies . . . . . . viii 25

Heurteloup's percuteur, for stone xii 415

Heusinger, Dr., his theory of development . . . . ix 526

on comparative pathology . xxiv 81

on the simia satyrus . . x 251

Hewes, Mr., his clairvoyant Jack . xix 459

Hewett, Mr., on a peculiar aneurism xxiv 323

on extravasations . . . xxiii 410

on omental sacs in hernia . xx 93

Hewson, William, his discoveries . xiv 486

works of . . . . . xxiii 251

Hey, Mr., his case of aneurism . xx 96

H— M—, Miss, Mr. Greenhow on the case of . . . . xix 429

Hiccup, or singultus, observations on xxiv 173

prolonged, treatment of . . xvii 420

treatment of . . . . xxiv 174

Hicks, Mr., his case of scalded glottis vi 270

Hidriotic fevers, remarks on . . v 290

Hidrosis, case of . . . . v 290

Hildanus, F., inventor of speculum auris iii 80

Hilles, Mr. M. W., on hernia . vi 203

Hill, Mr., on Lincoln Lunatic Asylum ix 153

on lunatic asylums . . . ix 144, 153

Hilton, Mr., on the laryngeal nerves vi 61

Himalayan Botany, Mr. Royle's . iii 205

mountains, botany, &c. of . i 215

cultivation on . . . i 345

Hindoo anatomy, account of . . xxiii 537

chemistry, ancient . . . xxiii 535

medical practitioners . . vi 508

writings, account of . . xxiii 525

medicine, ancient, remarks on . xxiii 543

antiquity of . . . vi 506

before Hippocrates . . vi 507

Dr. Royle on . . . vi 506

philosophy in . . . xxiii 533

nation, physical history of . xxiv 456

Hindoo notions on physicians . xxiii 534
observations on physicians . vi 508
pharmacy, notice of . . xxiii 536
practice of physic . . . xxiii 540
surgery, account of . . . xxiii 538
system of medicine . . vi 507
ancient . . xxiii 521
therapeutics . . . . . vi 507
women, puberty among . . xxii 289
Hindoos, cerebral affections rare in . xvii 155
Hindustani anatomical plates . . xxii 227
Hip, contracted . . . . . xvii 420
Hip-disease, flattening of buttock in iii 146
lengthening of limb in . iii 146
spontaneous dislocations of . xvi 486
luxation of, rules on . . xvi 489
luxations of, cases of . xvi 487
new bed for . xvi 486
Hip-joint, amputation at . xiii 173, xxii 113
anomalous dislocations of . xiv 37
artificial, on forming . xx 50
causes of diseased . . iii 169
congenital luxation of . xxi 403-6
contracted, treatment of . xiii 20
disease of, Chelius on . xx 114
contraction from . . viii 401
diagnosis of . iii 171, xx 115
frequent cause of . . xiii 169
lengthening of limb in . iii 171
length of limb in . . xxii 78-83
local remedies in . . iii 170
mercury in . . . . x 287
operation for . . xxii 84
treatment of . . iii 147, 171
use of seton in . . iii 171
dislocations of . . xiv 34-9
in children . xiv 31
fatality of injuries of . xi 461
Mr. Coulson on diseases of ii 231, iii 169
on pathology of . iii 170
Mr. Cox on amputation at xxii 111
Mr. Tyrrell on disease of . iii 146
on the seat of disease in . iii 170
operation for anchylosis of x 282
spontaneous dislocation of xiv 59
symptoms of diseased . iii 171
HIPPOCRATES, and ARTAXERXES . xix 122
HIPPOCRATES, and his writings . xxiii 92
birthplace of . . . . xvii 459
commentators on . . . xvii 461
descent and family of . . xvii 459
doubt of his existence . . xv 107
eulogy of . . . . . ii 530
genuine works of . . . xvii 466
his grumbling, in old age . xix 123
his physiology . . . i 380
his style . . . . . xvii 470
Littré's edition of his works . xvii 450
maxim of . . . . . xxiii 91
medical character of . . xvii 470
practice of . . . xvii 470
not the "Father of medicine" . xvii 459
on cures by fire . . . vii 63
HIPPOCRATES on the causes of disease xvii 469
on the medical opinions of . xvii 468
on the study of man . . vi 298
on typhus fever . . . xii 295
Peterson on his writings . xvii 450, 464
to the Abderites . . . xix 122
various editions of . . . xvii 452
works attributed to . . xvii 459
of, by M. Littré . . xxiii 253
Hippocratic collection, classification of xvii 467
critics on the . . xvii 462
M. Link on . . xvii 463
M. Littré on xvii 453, 460, 465
writings in . . xvii 466
writings, anatomy in . . xvii 465
Hippurate of soda and ammonia . xiii 263
Hippuric acid, and its tests . . xiii 263
in gouty concretions . xiv 52
in human urine . . xix 569
HIRSCH, Dr., on spinal neuroses . xxi 280
on thymic asthma . . . ii 237
Histological theory, basis of . . xxiii 137
Histology, and anatomy, special . xviii 223
M. Burggraeve on . . . xxiii 137
pathological, Gunsburg's . xxiv 118
History, and theory, medical, chairs of xxiii 106
medical, treatises on . . xxiii 86
of surgical operations . . xvi 46
HJORT, Dr., on radesyge . . xiii 461
HOBLYN, Mr., his Dictionary of Medical Terms . . i 517, xix 245
HOCKEN, Dr., his cases of phthisis xix 148-54
on amaurosis . . . . x 246
himself . . . . xix 147
hysteria . . . . xiii 520
inflammation of the retina . xx 510
the constitution . . xiii 520
the cure of phthisis . . xix 140
HODGKIN, Dr., Dr. Marx on . . xv 24
his lectures on health . . i 313, 362
his Morbid Anatomy iv 31, 55, xi 401
on adventitious structures . xviii 369
typhus fever . . xii 294, 311
calculus . . . . iv 375
preserving health . . xiii 519
the means of promoting health i 362
HOEFER, Dr., on platina . . xi 523
HÖEGH-GULDBERG, Dr., on delirium tremens . . . . vi 320
HOEVEN, Dr. Van der, on the Negro race xviii 372
HOFFBAUER, on the law in insanity xix 33
HOFFMANN, Dr. D., on medical jurisprudence . . . iv 489
HOFFMANN, Fred., his Physiology . i 386
on typhus fever . . . xii 295
HOFFMAN, J. and C., their medical system . . . . iii 451
Hog, variations in, when run wild . xxiv 59
Hogs, very subject to acephalocysts . xxiii 69
HOHENLOHE, Prince, on his cures . xv 423
HOHL, Dr., on obstetric exploration i 85
HOHNBAUM, Dr., on epigastric pulsation vii 511
HOLCHER, Dr., his Hanoverian Annals iii 460

HOLLAND, Dr. Calvert, as a physiologist xviii 304
his statistics of Sheffield . . xvii 528
on diseases of the lungs . . xviii 302
on moving powers of the blood xix 557
remarks on his style . . xviii 307
HOLLAND, Dr. H., his Medical Notes viii 482
on inquiry as to contagion . xxiii 313
Holland, epidemic cholera in, 1832-3 iii 485
medical degrees in . . . vi 280
literature of . . vi 283
military men in . . vi 282
present state of medicine in . vi 275
the medical profession in . vi 280
universities of . . . vi 275
want of institutions in . . vi 280
HOLROYD, Mr., on quarantine laws . xvi 290
HOLST, Dr., his tables of lunacy in Norway i 278
on medicine in Norway . . iv 541
the health of prisoners . xii 498
the Norway prisons . . xvii 88
tumour of the brain . . vi 226
Holly, on its use in ague . . xx 238
HOME, Dr. John, his Infirmary Report v 601
HOME, Dr. obituary of . . . v 628
HOME, Sir Everard, and Hunter's museum iv 86
his Life of Hunter . . . iv 75
his microscopy . . . xiv 488
Home station, diseases on the . . xvii 313
Homesthetic power in organisms . xxiv 189
Homicidal insanity, principle of . x 157
remarks on . xvi 83, 91, 109
lunatics, Mr. Taylor on . . xxi 486
mania, from impulse . . xvi 109
jurisprudence of . x 138-47, 167
two forms of . . . xx 27
monomania, cases of . . xix 40
remarks on . . . xxiv 240
Homicide, maniacal and sane, diagnosis of xvi 91
Mr. Watson on . . . v 171
Homœopathic doctrines, Dr. Symonds on xxii 563
hospital at Vienna . . xxi 240, xxii 570
practice, apology for . . vi 197
regimen, remarks on . . xxi 232
sectaries in Austria . . xxii 567
treatment, cases cured by . xxi 245
cases illustrating . . xxii 574-92
Editor's remarks on . . xxii 592
works, cases in, remarks on . xxi 518
Homœopathists, contentions among xxii 568
war among the . . . xxiii 610
Homœopathy, admissions in respect to xxi 250
and allopathy, by the Editor . xxi 502
a notable fallacy in . . xvii 222
aristocratic patronage of . . xxi 535
changes in the practice of xxii 565, 567
claims and importance of . xxi 238
correspondence on . . . xxii 243
cry against the Review on . xxiv 594
cure of diseases by . . . xxi 239
discovered by Shakspeare . xv 129
Dr. A. Combe on . . . xxi 520
Dr. Balfour's report on . . xxii 567
Dr. Bardsley on . . . vii 540
Dr. Bartlett on . . . xxii 237
Homœopathy, Dr. Gunther's estimate of i 191
Dr. Sankey on . . . xxiv 215
experimentum crucis on . . xxi 251
in Germany, present state of . xxii 565
manipulations in . . . xxi 236
mode of testing . . . xxiii 264
Mr. Walker's explanation of . xiii 209
objections to . . . . xxi 234
origin and history of . . xxi 227
prize essay on . . . iii 287
rationale of . . . . xxi 228
real principle of . . . xxi 524
the French Academy on . . iii 288
triumphs of, explained . . xxi 255
true estimate of . . . xiii 210
Homology and analogy, on the terms xxiii 475
Honduras, health of troops in . viii 214
medical statistics of . . viii 214
Honeycomb tissue of the eye . . vi 207
Hongkong, fever at, account of . xxii 182
cause of . . . xxii 183
HOOD, Mr., on diseases of children . xx 205
Hoofs, minute structure of . . ix 511
Hook, in operation for squinting . x 585-6
Mr. Tyrrell's, for artificial pupil xi 87
HOOKE, Dr., his discoveries . . xiv 483
HOOKER, Dr. J. D., on alcoholic drinks xxiv 532-4
HOOKER, Dr., on respiration . . vii 263
HOOPER, Dr., on the climate of Jersey iv 494
HOOPER, his Medical Dictionary . vii 526
his Vade-mecum, new edition of xiv 217
Hooping-cough, acetate of morphia in ii 178
alum in . . . xxiii 424
carbonate of iron in . vi 222
cerebral theory of . . vii 212
collection of facts on . xx 363
complications of . . vii 212
cure of, by fever . . xx 363
diagnosis of . . . xvii 296
Dr. Roe on . . . vii 210, 212
emphysema from . . xvii 296
essential nature of . . vii 211
endermic treatment of . ii 178
fumigations in . . i 574
hydrocyanic acid in . vii 213
inflammatory theory of . vii 211
many remedies tried in . ii 178
nervous theory of . . vii 211
opinion of Hufeland on . vii 211
pleuritis with . . vii 213
report on . . xvii 559, xx 557
subcarbonate of iron in . vii 283
treatment of . . . xx 558
HOPE, Dr., his character . . xii 287
his work on the heart . . xii 289
memoirs of his life . . . xiv 532
obituary of . . . . xii 286
on chronic pleurisy . . xiii 364
on diseases of the heart . . viii 448, 466
on inflammation of the brain . xii 100
on rheumatism . . . iii 555
sketch of his life . . . xii 286
Hôpital des Enfans, great mortality in i 497
in Paris . . . xi 198

Hôpital des Enfans Malades, in Paris vii 94
deaths in . xii 352
Hops, a pillow stuffed with, hypnotic xvii 110
Horaczek, Dr., on bilious dyscrasy xxii 311
Horæ subsecivæ, by Dr. Fletcher . ii 304
Horn and gelatine, formation of . xiv 513
Horn, developed in the human skin xx 87
Horner, Dr., his anatomy of cholera i 236
his special anatomy . . xviii 223
Horny excrescences on eyelids . xii 248
Horse, belladonna harmless to the . iv 130
buttermilk fatal to the . . iv 130
diaphoretics ineffectual in . viii 492
foot of the . . . . . xi 224
general notion shown by a . xi 92
section of muscles of eye of . xii 251
venereal disease in . . . xii 494
vesicular variolous disease in . ix 79
Horses' flesh, use of, as food . . xvii 16
Horses, roaring or cornage in, cause of iii 29
Hosack, Dr., biographical notice of ii 306
obituary of . . . . . ii 306
Hoskins, Dr., on vesical calculi . xv 537
Hospice des Enfans Trouvés, account of i 281
des Ménages, account of . . i 281
Larochefoucauld, account of . i 281
d'Orphelins, account of . . i 281
St. Michel à St. Mandé . . i 281
Hospices for the incurable, at Paris . i 281
old, in Paris . . i 281
Hospitallers, orders of, account of . xiii 291
Hospital gangrene, remarks on . xi 320
practice, Dr. Aldis's introduction to i 490
pupils, on examination of . ii 478
reports, Guy's and St. Thomas's i 543
St. Thomas's, by Mr. South i 203, 208
Hospital, for insane, at Rome, bad . i 498
at Sonnenstein i 499
foundling, of Moscow . . i 606
in Moscow, by Peter the Great i 603
la Pitié, in Paris, notice of . i 212
military, in Russia, 1706 . i 603
of incurables, at Geneva, bad . i 498
the homœopathic, at Vienna . xxii 570
the Seraphim, in Stockholm . xxii 459
Hospitals for children, insalubrity of xii 352
for the insane . . . . xiii 213
foundling, history of . . xiii 290
treatises on . . . . xiii 289
Dublin, statistics of . . ii 185
in China, establishment of . xii 290
in Denmark, account of . . iii 565
in Ireland, statistics of . . ii 185
state of . . . . . xiii 515
in Paris, account of . . i 281
in Prussia, account of . . i 303
in Russia . . . . . iv 272
in St. Petersburg . . . iv 277
naval, lectures at . . . xviii 221
on appointments to large . iv 287
Hossard, and his lever-belt . . xvi 348
and the French Academy . xvi 348
Hot alkaline mineral waters . . xiv 347
Hot and cold applications, Mr. Key on iii 121
saline mineral waters . . xiv 346
sulphurous mineral waters . xiv 346
water, on warming buildings by xix 17
Hôtel Dieu, account of . . . i 281
in Paris, notice of . . i 212
Hottentots and Bushmen . . xxiv 450
remarks on . xxiv 77
Hottentots, descriptions of . . xxiv 451
Mongolian origin of . . xxiv 452
Hour-glass contraction, by os uteri intern. ii 85
W. J. Schmitt on . i 112
Hourmann, and Dechambre, on pneumonia of the old . . iv 501
Hourmann, M., on pneumonia of the old ii 543
on respiration of the old . . i 553
on the structure of the lungs . i 233
Housemaid's knee, or ganglion patellæ iii 151
House-pupils in French hospitals . i 494
Houses of parliament, lighting of . xix 19
ventilation of . . xix 18
Houses, on warming and ventilating xix 1
remarks on the site of . . i 353
Houston, Dr., his catalogue . . xi 513
on a fœtus without heart or lungs ii 596
Howard, Dr. B., on deficiency of food viii 524
Howship, Mr., on fracture of neck of femur ii 159
Hufeland, Prof., obituary of . ii 613
on nature and art in cures . xxiii 592
on nux vomica in dysentery . i 256
his medical society of Berlin . ii 579
Hughes, Dr., his cases of diseased lungs xv 430
his Manual of Auscultation . xxi 281
on abdominal effusion . . xiv 126
hydrocephalus . . . xii 346
incipient phthisis . . xii 331
malignant disease of lungs . xiv 131
paracentesis thoracis xviii 343, xxiii 419
pneumonia . . . xvi 320
the location of phthisis . xvi 317
Hull, Dr., his style, and quotations xvii 204
on determination of blood xvii 200, 205
on diseases of the eye . . xi 221
Human contrivances, imperfection of xvii 449
embryo, on the brain of the xxii 497, 502
flesh, used as food . . . xvii 16
frame, observations on the . xii 8
life, on the renewal of . . xvi 208
race, adaptiveness of, to variation xxiv 67
original seat of the . . xxiv 479
primary source of the . xxiv 479
races, common origin of . . xxiv 441
difference of pelvis in . xxiv 78
one species and family . xv 183
physical diversities of . v 543
specific unity of . . xxiv 478
unity of origin of . . xxiv 50
variations in . . . xxiv 57
Humbert, M., on venereal diseases of skin . . . . . x 331, 420
Humble-bee, variable heat of . . xii 148
Hume, David, remarks on . . xii 150
Humeral artery, wounds of, in bleeding xx 48

Humeral dislocation, dissection of a xii 455

incision in old . . ix 555

dislocations, on incomplete . xii 454

luxation, myotomy in . . xiii 21

supra-condyloid process . . xix 571

Humerus, case of luxation of . . iv 256

congenital dislocation of . . x 566

dislocation of, backwards . ix 391

excision of head of the . . xii 545

luxated, a mode of reducing . vi 163

new mode of reducing . iii 241

luxation of, reduction of . . vi 231

singular dislocation of . . xv 402

ununited fracture of, cured . vi 69

Velpeau on luxations of . . v 569

Humidity, influence of, on diseases . xx 104

Humility, medical, observations on xxii 161

Humoral doctrines, remarks on the xvii 473

pathology, Dr. Paine on the . xi 392

remarks on the . . xv 369

Humour, vitreous, structure of the . xxii 286

Humphreys, Mr., his electro-physiology xvii 246

Humus, and humic acid . . . xi 437

carbon of plants not from . xi 437

Huenefeld, Dr., on chemistry and medicine . . . . . xvi 145

Hunger and thirst, nature of . . xvii 37

Hunger, Brachet's experiments on . ii 370

Dr. Alison on . . . vi 180

Dr. Beaumont's theory of . vi 179

Dr. Combe's theory of . . vi 180

its relation to waste of body . ii 371

nerves of sensation of . . xi 518

not caused by gastric juice . ii 136

source of the sensation of . iv 422

stomach and brain in relation to ii 370

vagi nerves in relation to . viii 268

Hunt, Dr. E. K., his translation of Esquirol . . . . . xx 520

Hunt, Dr. H., on cancrum oris . xviii 367

on tic douloureux . . . xviii 56

Hunt, Mr. R. T., his operation in ptosis x 26

on the muscles of the eye . iv 477

Hunt, Mr. T., on diseases of the skin xxiv 280

on the use of arsenic . . vii 151

Hunter, Dr. W., a deathbed saying of iv 191

on dysentery . . . . . xxiv 340

a letter of, to Cullen . . iv 190

as a lecturer . . . . . iv 190

his commentaries . . . . iv 191

his museum, &c. . . . . iv 191

his rise and progress . . iv 189

his writings . . . . . iv 190

on infanticide, faults of . . iv 88-90

on the gravid uterus . . xvii 530

Hunter, John, and Cuvier, remarks on . . . xv 195, 201

Hunter, and his museum, observations on xv 195

Hunter, John, and the magnetiser viii 493

and the giant O'Brien . . iv 83

and William, compared . . iv 77

wars of . . iv 78

Hunter, John, as a lecturer . . iv 80

at Oxford . . . . . iv 77

Hunter, John, characteristics of . vi 191

edition of his works . . iii 486

eloquent reference to . . vii 498

first investigated constitutional irritation . . . . . ii 1

his admirable researches . . iii 447

anticipations . . . vi 192

birth and pedigree . . iv 75

coming to London, &c. . iv 77

death, at St. George's . iv 85

disputes, at St. George's . iv 84

experiment on freezing . v 50

experiments on arteries . viii 459

"family property" . . iv 76

heart, after death . . iv 85

humble early life . . iv 76

ideas of inflammation . viii 183

industry and sagacity . xv 217

lectures, by Mr. Palmer . v 46

letters to Jenner . . iv 81

life and works . . . iv 75

mode of investigation . iv 80

museum . . . iv 80, 81, 86

and Mr. Pitt . . iv 86

and Sir E. Home . iv 86

preparation of insects . xv 215

punctuality . . . iv 81

pursuits and practice . iv 78, 79

speculation on freezing . iv 82

treatise on syphilis . . v 2, 30

lectures of, remarks on . . v 46

life of, by Dr. Adams . . iv 75

Jesse Foote . . iv 75

Sir E. Home . . iv 75

Mr. D. Ottley . . iv 75

on associated actions . . v 522

his style . . . . vii 418

inflammation vii 417, 420, viii 169, 171

the action of vegetables . v 489

animal economy . . vii 523

blood, &c., history of . vii 418

remarks on . . i 388

fifth pair of nerves . xv 213

growth of bone . . xxiv 399

life of blood, remarks on i 388

nervous system . . xv 211

peculiar merit of . . xxiv 217

spinal cord . . . v 528

vascular system . . viii 171

vital principle . . v 47

union by first intention . vii 432

remarks of Professor Owen on xv 217

singular complaint of . . iv 80, 82

sitting for his portrait . . iv 83

traits of his character . . iv 83

works of, by Mr. Palmer . v 46

Hunterian Museum . . . iv 86

additions to . . xv 196

arrangements of . xv 202

catalogue of . . xv 195

new, engravings in xv 199, 215

new indexes to . xv 200

Dr. Marx on the . xv 22

former catalogues of xv 198

Hunterian Museum, Home's synopsis of xv 197
invertebrata in . xv 201
preparations in . xv 202
purchase of . . xv 196
oration for 1837 . . . iv 189
Mr. Arnott's . . . xv 536
Mr. Green's . . x 545, xxiv 216
Mr. Travers's . . . vi 191
Huschke, his anatomy of the viscera xx 419
Huss, Dr., his report on diseases xxii 459
Hutchinson, Mr. Cop., on calculus in seamen . . . . vii 141
Hutchinson, Mr. John, on capacity of lungs . . . . . xxiv 327
on respiration . . . xxiv 167
Hutchinson, Mr. W. B., on revaccination vii 581
Hutchinson, Rev. E., on tic douloureux vi 511
Hutchison, Rev. Dr., quotation from xix 473
Huxham on typhus fever . . xii 295
Hybernating animals, loss of heat in xiv 503
Hybernation, and sleep, Hunter on vii 524
causes and nature of . . xxiv 409
Dr. Barkow on . . . xxiv 408
temperature in . . . xxiv 408
Hybrids, capability of procreation in iii 373
in plants, fertility of . . iv 29
remarks on . . . iv 29
Hydatid cyst on prostate gland . xxiv 328
remarkable, on diaphragm xxiv 180
pill-box, description of xvii 195
Hydatid (Echinococcus), researches on xvii 194
of liver, a rare species of . xi 463
tapped . . . . vii 154
Hydatids, and moles, origin of uterine iv 98
death of, in swine . . . xxiii 59
echinococci contained in . xvii 196
five orders of . . . . xx 406
free in the blood . . . xx 412
in criminal abortion . . iv 98
inflammatory origin of . . xxiii 69
in man, account of . . xvii 197
inoculation with ova of . . xx 413
in relation to legal medicine . xiv 211
on the nature of . . . xxiii 58
never cause tubercles . . iv 216
of brain . . . . . xx 408
case of . . . . . xx 409
of spinal cord and liver . . xx 411
of uterus in virgins . . xviii 22
Lisfranc on . . . xviii 22
or acephalocysts, remarks on . xvii 194
origin of, from without . . xx 413
pulmonary, case of . . xx 253
therapeutics of . . . . xx 415
uterine, Dr. Montgomery on . xiv 211
Hydatis spuria, account of . . xx 407
Hydra, the, all stomach . . v 489
Hydra viridis, structure of . . xxiv 134
Hydragogue cathartic for dropsy . xxi 370
Hydrargyrus c. creta, good effects of viii 439
Hydrarthrosis, congenital . . xxi 397
emetic tartar in . . vi 224, xi 247
iodine injections in . xviii 89, xxii 71
Hydrated oxide of iron an antidote to arsenic . . . . i 572-3
for arsenic . iv 237
sesquioxide of iron . . xiii 74
formula for . xiii 74
tritoxide of iron, how made . i 574
Hydraulic experiment by Dr. Williams xvii 492
Hydraulics, vital, M. Magendie on viii 328
Hydrencephalocele, case of . . xiv 246
Hydriodate of iron very easily decomposed ii 210
of potash, action of . . v 166
adulteration of . . v 165
dose of . . . . v 166
formula for . . . viii 597
in ascites . . . iii 438
in syphilis . . v xx, vii 245
poisoning from . . x 588
Hydrocarburets, nature of . . xi 133
Hydrocele, acupuncture in . . v 279, 607
acute, M. Velpeau on . . xii 452
chronic, M. Velpeau on . . xii 453
complicated with hernia . xxii 203
congenital, diagnosis of . . xvii 71
injection of . . . xvii 72
cure of, by incision . . xvii 69
by caustic . . . xvii 70
detecting transparency in . xvii 68
encysted, Mr. Liston on . xviii 369
inequalities of surface in . xvii 68
injection in . . . . xvii 70
injection of chlorine gas in . ii 267
of tinct. iodin. in . . xii 453
liquid of, coagulation of . . xxi 542
M. Baudens' treatment of . xvii 71
Mr. Curling on . . . xvii 66
Mr. Travers's operation for . iii 560
of spermatic cord . . . xvii 73
of the hernial sac . . . xvii 73
of the testis, encysted . . xvii 72
place for tapping . . . xvii 69
radical cure of . . . xii 453
spontaneous disappearance of . xvii 68
treatment of . . . . xvii 69
after injection in . . xvii 71
by acupuncture . . iii 559
Hydrocephaloid cases in children . iii 325
Hydrocephalus acutus, case of . xxii 403
Hydrocephalus, accidental cure of . v 565
acute, Dr. Davis on . . xi 151
Dr. Jahn on . . . viii 426
inflammatory . . . xi 152, 155
M. Rilliet on . . . xxiii 302
report on . . xvii 551, xx 554
theories of . . . xi 152
tubercular theory of . xi 154
a peculiar cause of . . . x 559
bloodletting in . . . xi 156
calomel in . . . . xi 157
case of cure of . . . ix 232
cathartics in . . . . i 20
caused by tubercles . . xii 229
causes of . . . . xi 155
caution on pressure in . . xvii 554

Hydrocephalus, chronic, case of . xiii 543
report on . xvii 554, xxiii 303
cold affusion in . . xii 559, xiv 206
corrosive sublimate in . . xxi 471
cured by compression . viii 560, ix 231
diagnosis of . . xii 347, xiv 205
discharged by the ear . . xiv 237
Dr. Bennet on . . . xv 539
Dr. Hughes on . . . xii 346
Dr. Mauthner on . . . xxi 386
Dr. Sims on . . . . iii 311
Dr. Vrolik on . . . xii 228
eruption in . . . . xi 154
formative stage of . . xi 154
general bloodletting in . . i 20
inflammatory stage of . . xi 154
in parturition . . . xii 480
youth . . . . . xx 260
ipecacuanha liniment in . xvii 554
its relation to scrofula . . xi 424
M. Berton on . . . xv 324
Mr. Griffith on . . . i 535
Mr. Mayo on . . . iv 246
paracentesis in . . viii 53, xii 119
pathology of . . . . iii 310
proximate cause of . . xi 155
remarks on . . . . iii 304, 310
remittent fever in . . . xi 154
report on puncture in . . xvii 555
results of puncture in . . xiv 256
spontaneous cure of . . xiv 237
state of pupil in . . . xi 154
success from iodine in . . xvii 551
symptoms of . . . . xiv 205
tapping the head in . . vi 265
third stage of . . . xi 154
treatment of . . . i 20, xi 156
tubercles in . . . . ix 218
Hydrochloric acid in air . . xix 14
in gastric juice . . xvi 148
stomach, source of . xi 332
Hydrocyanic acid, action of . . viii 355
antidotes for . . xviii 553, 554
detection of . . xvi 78, xx 337
from the urine . . xx 67
history of . . . xiii 57
in hooping-cough . . vii 213
lotion of . . . xv 390
mode of action of . . xx 318
Mr. Pereira on . . viii 358
poisoning, shriek in . xx 325
poisoning with iv 533, vii 572, xx 333-8
power of action after taking
much . . . xviii 559
preparation of . . xiii 58
properties of . . . vii 572
remarks on . . iv 109, xvi 191
tolerance of . . . vii 213
trials for poisoning by xviii 559, 560
Hydrocyanic ether, account of . ii 208
Hydrogen, as an element of food . xvii 2
assimilation of, by plants . xi 439
the acidifying principle . . xi 140
Hydrometra, case of, by Dr. Ashwell xix 355
or uterine dropsy . . xv 105, xviii 21
Hydronephrosis, diagnosis of . . viii 125
M. Rayer on . . . . xv 484
Hydropathic bathing and sweating . xii 191
establishments, on distinct . xxii 430
patients, diet of . . xxii 437, 442
practice, appreciation of . . xxii 440
stinking perspiration . . xii 192
system, estimate of . . xii 193
treatment, details of . . xxii 437
in fever . . . xviii 195
M. Bonnet on . . . xxii 66
the Editor on . . . xxiii 269
Hydropathy, and Hydriatics, origin of xii 189
boils and eruptions in . . xii 192
calm investigation of . . xxii 429
cry against the Review on . xxiv 594
diseases treated by . . xiv 432
Dr. Freeman's system of . xvi 471
Dr. Seymour on . . . xxiv 149
Dr. Symond's remarks on . xxii 562
effects of, on the organs . . xxii 452
exercise and sweating in . . xxii 438
important impressions on . xxii 457
Mr. E. Wilson on . . . xxi 204
note on . . . . . . xv 463
observations on . . . xxi 254
on recommending . . . xiv 437
or the cold-water cure . . xiv 422
present estimate of . . xiv 433
regimen in . . . xiv 430, 436
selection of cases for . . xxiv 150
therapeutic qualities of . . xxii 454
the three modes of . . xiv 433-6
tranquillizing effects of . . xxii 453
treatises on . . . . xxii 428
Hydro-pericardia, Dr. Crichton on . i 259
Hydropericardium, active and passive ii 331
diagnosis in . . . ii 332, vi 132
measure of fluid in . . ii 332
sound of . . . . . vi 132
Hydrophobia, action of the poison in xvi 420
belladonna as a preventive of . i 25
cantharides a remedy in . i 25
cases of . . . . . vi 268
convulsions from idea of water in xix 302
cures of . . . . . xii 108
Dr. Williams on . . . xiii 90
effect of civilization on . xviii 244, 250
effects of reflected light in . xix 301
exclusion of light in . . xi 237
experiments on . . . iv 240
fatal closure of glottis in . xvi 170
from a healthy dog . . xiii 53
bite two years before . xix 375
man to man . . . xiii 52
history of . . . . xiii 50
idiopathic, case of . . . xiii 559
in animals . . . . xxiv 90
in man . . . . . xiii 51
in the cat . . . . xiii 51
in the dog, two forms of . xiii 50

Hydrophobia, irritability in . . xi 454

never originates in man . . i 372

observations on . . xi 237, xx 264

on extirpating parts in . . xiii 92

origin and propagation of . xiii 93

pathology of . . . . xvii 422

period of incubation of . . xiii 51

prevention of. viii 53, xiii 92, xvii 422

preventive treatment of . . i 25

reflex action in . . . xix 301

stages of . . . . xiii 52

statistics of . . . . xvii 422

transmission of . . . xi 229

in dogs . . . xiii 52

to man . . . xiii 52

treatment of . . . . vii 270

use of Indian hemp in . . x 227

Hydrophobic experiment of Magendie xiii 53, 94

inoculation of dogs . . xiii 52

poison, on the action of . . xiii 90

Hydrophosphoric acid aids putrefaction ii 396

Hydrophthalmia, case of . . xvii 236

Hydro-pneumo-pericardium . . xx 255

Hydrops articuli, cause of . . x 332

cured by ague . iii 511

Hydrosis, observations on . . xix 235

Hydrostatic test, Barzellotti on the ix 49

case in relation to . . v 583

of infanticide ii 411-14, v 180, ix 47, xvi 69

value of . . . . iv 90

Hydrothorax, arthritic, of old age . xvii 115

Dr. Chapman on . . . xxi 370

prescription for . . . xxi 371

seldom a primary disease . i 20

Hydruria, in the horse, acetic acid in xxiv 86

nature and treatment of . . vii 159

Hygienic, parliamentary, measures . xv 330

treatment of disease . . xxi 264

Hygiene, and therapeutics, remarks on iii 458

attention to, abroad . . i 358

urged on practitioners . i 358

Dr. Lévy on . . . . . xix 51

Elements of, by Dr. Dunglison i 313, 344

general, Dr. Motard on . xiv 446, 461

on cultivation of . . xvii 403

in France and Germany . . xiv 446

in general, Mr. Chadwick on . xviii 207

importance of a knowledge of i 314, v 382

of the study of . . x 188

instruction of the poor in . i 361

in treatment of disease . . xxi 506

lectures on, by Dr. Kilgour . i 313, 352

mental, Dr. Sweetser on . xvi 511

military, remarks on . xiv 382, 389

works on . . . xix 377

M. Royer-Collard on . . xviii 153

neglect of, in England . . i 359

of Great Britain . . . xviii 492

of the consumptive . . xx 108

philosophy of . . . xiv 446

popular works on . . . xviii 115

practical obstacles to . . xviii 207

Hygiene, public, a knowledge of . v 452

imperfection of . . xiv 460

importance of . . . xviii 493

M. Duchatelet on . v 447, xi 1, 13

medical witnesses on . v 447

observations on . . v 447

study of . . . . v 449

recent popular works on . v 380

relation of clothing to . . i 332

reviewers' labours on . . xix 51-2

science of, in England . . xiv 446

Hygrometer, in relation to health xxiv 101, 113

new, simple . . . . xxiv 283

Hygrometers, natural, in Africa . xi 366

Hygrometrical tables, Mr. Glaisher's xxiv 283

Hymen, danger from dividing the . xi 179

persistence of, in labour . . xxiii 281

Hyoscyamus, fecula of . . . iii 229

in diseases of the eye . . x 8

use of, in insanity . . . xxii 57

Hyper-catharsis, convulsions in infants from . . . . . xx 364

Hyperceneses of the nerves . . xvii 420

Hyper-chemosis, signification of . xvi 196

Hyperemia, or congestion, Mr. Mayo on iii 57

Dr. Williams on . . . xvii 488

of brain in old age . . xvii 109

table of varieties of . . xvii 489

Hyperesthesiæ, cutaneous . . xvii 410

of pneumogastric . . . xvii 414

Hyperesthesia of central organs . xvii 416

psychica . . . . xvii 418

Hyperinosis of the blood . . xviii 257

Hyperostosis, of skull, puerperal . xix 392

Professor Albers on . . xvi 382

with osteitis . . . . ii 537

Hypertrophy, and organized lymph, diagnosis of . . . ii 458

Andral's error in the term . ii 456

concentric, nature of . . ii 459

Dr. Canstatt on . . . xiii 329

Dr. Carswell on . . . ii 456

from congestion, remarks on . xvii 494

nature of . . . . ii 457

of brain in children . xx 555, xxi 386

of cervix uteri . . . xix 356

of heart, acute form of . . ii 335

Dr. Latham on . . xxiii 180

in children . . . iii 228

signs of . . . . ii 334

three kinds of . . ii 333

treatment of . . . ii 335

of mammary gland . . iv 224

of skin, hereditary . . . ii 459, 568

size of heart constituting . i 432-3

treatment of . . . . xiii 330

varieties and causes of . . ii 457

Hypinosis of the blood . . . xviii 257

Hypnotism, Mr. Braid's process of . xix 476

Hypochondria, Dr. Michéa on . xx 375

essay on . . . . xviii 162

its connexion with gout . . xvii 418

pathology of . . . xvii 418, xx 26

Hypochondria, Professor Romberg on xvii 418

psychological . . . . xx 375

treatment of . . . . xvii 419

Hypochondriasis, cerebral origin of xx 366

causes of . . . vii 27, xx 369

pathology of . . . xx 365, 370

phenomena of . . . xx 367

remarks on . . . . xii 67

seat of . . . . . xx 370

treatises on . . . . xx 364

treatment of . . . . xx 371

Whytt on atrophy in . . ii 464

Hypoglossus nerve, functions of . xviii 138

origin of . . . xviii 136

report on . . . xxii 284

Hypopium in corneitis . . . vii 218

Hypospadias, Hunter's case of . viii 17

operation for cure of . . xxi 314

Hyposthenisant medication, remarks on xxi 161

Hypothesis, and law, relation of . xx 142

in physical science . . . xx 143

Hypotheses and theories, use of . xxiii 103

Hysteria, accelerated pulse in . xii 107

and epilepsy, diagnosis of . xvii 134

and gout relation of . . xii 73

cases of . . . . . v 601

cause of . . . . . xii 123-5

closure of glottis in a case of . xvi 170

condition of the ovaria in . xii 72

Dr. Davis on . . . . iii 401-2

Dr. Laycock on . . . vii 282

Dr. Roots on treatment of . iii 144

Dr. Watson on . . . xvii 134

in men, cases of . . . xiv 247

in relation to mind . . xv 420

irregular forms of . . . xii 77

laxity of tissues in . . . iv 136

marriage as a remedy for . xx 31

mercury and bleeding in . iii 402

mischiefs of 'nimia diligentia' in xviii 64

morbid cunning in . . . xvii 421

Mr. Prichard on . . . vi 512

M. S. Pinel on . . . . xx 31

no traces of, in death . . iv 136

objections to the term . . xii 122

origin of . . . . . iii 177

pathology of . . . . vi 512

Dr. Hocken on . . xiii 520

philippic in relation to . . vii 516

political evils of . . . xx 33

pretty and small medicines in xvii 421

Professor Romberg on . . xvii 421

relation of, to mesmerism xix 436, 447

simulating peritonitis xii 107, xvii 135

phrenitis . . . xii 107

spinal, bad practice in . . iv 136

treatment of . . . . iii 402

use of creosote in . . . ii 169

heat in . . . . xix 186

physiognomy in . . xii 108

vascular relation of . . xii 66

vomiting in, remarks on . ii 200

with salacity, case of . . xx 32

Hysteric abdominal pain, pulse in . xii 107

diseases, from gouty habit . xii 69

ischuria in females . . xii 122

symptoms in the male . . xx 32

trance, cases of . . . xix 437-9

Hysterical affection of the eyes . xiv 246

of vocal organs . . xviii 367

affections, Dr. Laycock on . xii 60

from spine . . . iv 136

local, Sir B. Brodie on . iv 135

sulph. cupr. in . . iv 137

treatment of . . . iv 137

females, urinary tricks of . xi 362

pains, remarks on . . . ii 201

paralysis, case of . . . iii 143

retention of urine . . . iv 136

sopor, hot poker for . . xvii 202

young ladies, piety of . . xx 33

Iatraliptic method, remarks on the . v 343
Iberians, ancient, present descendants of xxiv 464
Ices, eating, monitory remark on . ii 388
Ice, internal use of, in gastritis . ix 476
success of, in aphonia . . v 439
to the head, in burns, recommended i 496
treatment of aneurism by . v 568
use of, in puerperal fever . iv 518
inflammations . xiii 120
peritonitis . xiii 108
Iceland moss, bread of . . . xiii 66
Iceland, trismus nascentium in . viii 486
Ichthyosis cornea, case of . . iv 229
Ichthyosis intra-uterina, cases of xx 549, xxiii 298
Icterus, Dr. Horaczek on . . xxii 311
neonatorum, remarks on . . xx 357
report on . . . xx 553
symptoms of . . . . xxii 312
treatment of . . . . xxii 317
Ideagenic and kinetic substrata . xix 308
Ideal period, in Germany, remarks on iii 450
Idea, an habitual, becomes a motive . xxiv 226
Ideas, and notions, distinction of . xxiv 11
association of . . . xix 311
cerebral, feelings, sensorial . xxii 512
inscribed, and expectations . xxiv 198
material, action of, on the brain xxiv 192
of, on the nerves . xxiv 193
Unzer's doctrine of . . xxiv 186
simple, and active principles . xxiv 226
Ideler's edition of Dietz's Hippocrates xvii 451
Idiocy and imbecility . . . x 148
M. Pinel on . . xx 25
Idiocy, curability of . . . xxiv 3
definition and symptoms of . xxiv 2, 4
from blow on the head . . x 277
in various countries, remarks on i 276
its great prevalence in Turkey . iv 391
M. Marc on . . . . . x 167
Mr. Colquhoun on a case of . vi 122
or fatuity, Scottish law of . vi 123, 125
Idiopathic and symptomatic disease xxiv 305
Idiopts . . . . . . xvii 415

Idiosyncrasies, caution on . . x 46
Idiot boy, interesting case of an . xix 294
Idiot, appearances in the brain of an vii 32
head of an . . . . ix 208
in labour, gnawing the cord . xix 309
Idiots and cretins, shape of heads of viii 19
Idiots, arrangement how taught to . xxiv 9
coercion of . . . . xxiv 17
education of . . . . xxiv 1, 3
applied . . . . xxiv 13
ear in . . . . xxiv 7
in arithmetic . . . xxiv 12
in drawing and reading . xxiv 10
memory of . . . xxiv 12
muscular system of . . xxiv 4
sight of . . . . xxiv 9
speech in . . . xxiv 7
taste and smell in . . xxiv 6
touch in . . . xxiv 6
effect of fever on minds of . iv 433
in the Bicêtre, Dr. Conolly on . xxiv 21
education of . . xix 292
moral treatment of . . xxiv 14
Iganie, cholera after the battle of . xxiv 327
Ileitis, diagnosis of . . . ix 478
Ileo-cæcal abscess, case of . . viii 279
Ileum and bladder, case of fistula of xx 101
Ileum, inflammation of . . . xi 420
perforation of, from without . xx 221
recovery after . xx 243
portion of, passed per anum . ii 601
separation of part of the . . xii 540
softening of, in typhoid fever . xii 26
Ileus and constipation, cases of . xx 242
Ileus, an extraordinary, fatal case of iv 168
a nondescript cause of . . iv 168
belladonna clysters in, cases of iv 220
case of, cured by inflation . ix 221
cause of obstruction in . . iii 75
from hypertrophy of pancreas . xii 538
from intestinal diverticulum . iv 168
from scirrhous degeneration . xv 98
Mr. Mayo on . . . iii 74
or iliac passion, remark on . xviii 313
paralysis, not spasm, in . . xxiv 311
Iliac, common, tying of, in dogs . xxii 107
external, case of ligature of . iv 151
Lisfranc on tying . . xiv 25
Iliac abscesses after delivery . . ix 460
arteries, aneurism of . . xxiv 421
artery, external, ligature of vii 154, xxiv 326
fossa, abscess in the right . ix 451
fossæ, statistics of tumours in . ix 458
tumours, diagnosis of right . ix 454
Illegitimate births in European states xiii 296
children, statistics of . vii 130, xxi 3
Illusions and hallucinations . . xx 22
Illusions, definition of . . . x 166
spectral, singular case of . . iii 261
Illustration, curious, by Mr. Travers ii 8
Illustrations of disease, by Dr. Carswell i 117,
ii 456
Illustrious name, remarks on an . iv 76
Images, by pressure of the retina . x 16
inverted and erect, in vision . x 14
use of, in educating idiots . xxiv 9
Imagination, morbid influence of . xv 419
severe cases cured by . . xxiii 267
Imbecile and helpless lunatics . xix 287
Imbeciles, examination of alleged . x 150
responsibility of . . . x 150
Imbecility, and idiocy, M. Pinel on . xx 25
Imbecility, Dr. Prichard on . . xvi 86
general state of mind in . . xxiii 235
medical evidence in . . x 149
jurisprudence of . . x 148
Miss Bagster's case of . . x 155
validity of deeds in . . x 147
Imbibition, and selective absorption xiv 564
Imbibition, experiments on . . xxiii 383
M. Magendie on . . . viii 311
observations on . . . xxiii 382
redness from . . . . viii 195
Imitation, influence of . . . v 518
in instruction of idiots . . xxiv 5
on the propensity to . . xxii 513
Imminence, morbid, Dr. Levy on . xix 57
Immoveable apparatus in fractures . vi 310
objections to . . vi 315
Impacted head, case of . . . ix 262
Imperforate anus, curious case of . xxiii 301
Impetigo, characters of . . . xv 389
diagnosis of . . . . xv 389
lotion for . . . . xv 390
M. Rayer on . . . . ii 152
of the scalp . . . . xv 117
causes of . . . xv 118
prognosis of . . xv 118
treatment of . . xv 119
sparsa, of scalp . . . xv 118
syphilitic, characters of . . xxiii 356
treatment of . . . . xv 389
Imponderable agents, operation of . xx 155
Impostors and quacks, patronage of xxi 533, 535
Impoverished blood, diseases from . viii 324
Impregnation, ages most susceptible of viii 51
by a syringe . . . . viii 17
in plants, remarks on . . iv 29, 561
of the mammifera, Mr. Jones on iv 527
ovaries five days after . . xvii 272
state of vesicle after . . iv 460
Impressions and conceptions, Unzer on xxiv 186
centric and peripheral . . xxiv 201
diffusion of . . xxiv 207
nervous, diffusion of . . xix 307
non-transmissible . . . xxiv 190
sentient . . . . xxiv 188
Imprisonment in the army, objections to xxiii 167
remarks on . xix 388
Inaction, muscular, bad effects of . v 145
Inanitiated frogs, blood and liver of xvii 350
persons, application of heat to . xvii 354
Inanitiation, death from, in diseases xvii 355
Inanition, effect of artificial heat in . xvii 354
effects of, on the blood . . xvii 350
the fat . . xvii 350

Inanition, effects of, on the heart . xvii 351

on the nervous system xvii 351

on the stomach . xvii 351

experiments on . . . xvii 344

fecal matter in . . . xvii 353

general symptoms in . . xvii 353

in fever, artificial heat in . . xvii 356

influence of age during . . xvii 346

in relation to heat . . . xvii 352

in the insane, gangrene of lungs from ii 547

loss in different organs by . xvii 350

of weight in animals by . xvii 345

the respiration in . . . xvii 353

Inappetency, stage of, in dementia . vii 10

Incas of Peru, notices of the . . x 479

observations on . . xxiv 382

Incendiary mania in young females x 170

monomania . . . . . xx 28

Incident motor nerves, Dr. Hall on xvii 175

Incision, Mr. Middlemore on . iv 256

on a particular mode of . iv 256

the proper line of . . xv 34

subcutaneous . . . . ix 554

Incisions, elliptical and crescentic . xx 44

for tumours, on the first . xv 34

on tension of skin before . xv 34

Incoherence, four degrees of . . vii 9

or dementia . . . . . vii 9

Incomprehension, stage of, in dementia vii 10

Incontinence of urine, belladonna in xxiii 376

cantharides in . . ii 549

in childhood . . . xx 559

treatment of . . . ii 555

Incorporation of the profession xix 204, 550, xxi 532

Incubation, heat of, M. Guyot on . xix 183

of insanity, period of . . xx 19

Incurable diseases, treatment of . xxiii 545

Indentation of os frontis, in infant . xiv 236

India, E., Company's workmen, deaths of xiv 113

army in, on statistics of . . xxi 379

causes of acute diseases in . ix 564

constitutions of the natives of. iii 352

convalescent retreats in . . i 345

diseases of, Dr. Geddes on . xxiii 106

spleen in . . . i 563

dysentery and hepatitis of . xxiii 146

on the health of Europeans in xii 423

huts of the poor in . . ix 236

immunity of from typhns fever (App. to vol.) xi 46

mortality of British soldiers in iii 49

of troops in, rate of . vi 150

Mr. Annesley on diseases of . xiii 347

remarks on phthisis in . . iii 330

rubber bandages . . . xii 252

pleximeter . . . iii 187

stone in bladder frequent in . iii 330

surgical diseases in . . xii 422

ungenial to the scrofulous . i 346

Indian Archipelago, animals of the . iii 370

on peopling of . . xxiv 472

drugs used by the Arabs . . xxiii 528

Indian hemp, efficacy of . . xvi 357

medical properties of . xviii 368

or hachish, Dr. Moreau on xxiii 217

Journal of Medical Science iii 329, xiii 347

medical edict, ancient . xxiii 522, 523

Medical Journal . . . iii 192

medical press, remarks on the . iii 329

medicine and surgery . . xix 72

native doctors, or hakims . xix 77

practice in cholera, Dr. Bene on i 15

physicians in Alexander's camp xxiii 529

Quarterly Medical Journal . . vi 260

system of medicine, ancient . xxiii 521

Indians, American, harmless excess of vi 179

North American, Mr. Combe on xv 58

sudatories, &c., of . . xxii 435

Indigestion, a common cause of . iv 62

changes in the nature of . vii 124

dietetical regimen in . . xvii 51

different origin of . . . vii 119

Dr. Child on . . . . xxiii 577

Dr. Philip's cases of fatal . ii 141-2

Dr. Philip on treatment of . ii 144

Dr. W. Philip on . . . xv 524

from cerebral affection, diagnosis of ii 141

excess of food . . vi 178

morbid sensibility . . vii 125

hygienic treatment of . . vii 478

inflammatory kind of . . vii 124

instructive cases of . . vii 120

investigation of . . . vii 120

irregular action of heart in . vii 128

on alkaline . . . . . ix 273

origin of . . . . . xxiv 579

salt and water in . . . v 390

Indigo, form of exhibition and dose of ii 245

its effects in epilepsy . . ii 244

manufacture of . . . xiii 77

physiological effects of . . xiii 77

possible division of a grain of . xi 138

virtues of . . . . . xiii 76

Indo-Atlantic nations, the skulls, &c. of xxiv 454

-Chinese peninsula, races of . xxiv 462

races, language of the . xxiv 463

-European nations . . xxiv 456, 459

-Malayan or Western Malays . xxiv 467

Indolent people, lesson to . . vii 382

tumours, origin of . . . ii 17

Induced muscular contraction . xxiii 407

Indurated cellular tissue of infants . xi 202

Induration, mistaken for malignity iv 172

of cellular membrane in infants ii 361

tissue in infants . xx 553

Induction, its real character . . xx 142

Inductive sciences, history of the . v 317

system, applicability of . . v 325

Infancy and childhood, on diet in . xxiii 299

Dr. A. Combe's treatise on . xii 228

early, diseases of, report on . xxiii 300

moral management of . x 241-51, 514

on the mortality in . . x 515

sources of disease in . . x 517

Infanticide, absurd English law of . vi 65

Infanticide, Barzellotti on . . ix 46

by strangulation . . . ii 415

case of . . . . . v 618

case of cranial fracture in . iv 97

alleged . . . . xiii 250

suspected . . . . vii 257

in relation to . . . . v 583

cases in relation to . . . vi 65

of, with remarks . . iv 521

causes of death in . . . . iv 95

Cummin and Schwörer on . iv 87

deaths in, in Ireland . . xviii 40

Dr. Taylor on . . . . xvi 309

Dr. W. Hunter's opinions on . iv 87-90

during birth . . . . . iv 94

ecchymosis on child in . . iv 100

effect of the tours on . . xiii 300

effects of civilization on . . xviii 241

emphysematous lungs in . . iv 91

English law of . . . . iv 94

form of chest in . . . . iv 90

fractures of cranium in . . iv 96

hydrostatic test in iv 91, 95, vi 64, ix 47, xvi 69

inflation of lungs in . . iv 92

in privies, remarks on . . vii 138

jurisprudence of . . . xvi 68

legal decisions in . . . . iv 93

malaria in relation to . . xviii 504

marks of violence in . . iv 96

medical evidence in . . . vii 139

obscure case of suspected . ii 416

on pulmonary tests of . . vi 66

Prof. Devergie on . . . . ii 405

remarks on a case of . . xiii 252

on proofs of . . . vii 259

respiration of child in . . iv 93

the lungs in relation to . . xvi 68

Infantile apoplexy, causes of . . iii 550

Dr. Kennedy on . . . iii 550

diseases, diagnosis of . . . ii 353

peculiarities of . . . xx 550

Tortual on . . . . ii 358

Vogel on . . . . ii 353

erysipelas, report on . . xvii 550

gangrene, Dr. Richter on . vii 470

life, epochs of, Vogel on . ii 355

mortality, report on . . xx 549

pneumonia, as to age . . vii 81

bloody froth in . . . vii 79

cough in . . . . vii 79

duration of . . . . vii 81

etiology of . . . . vii 82

lesions in . . . . vii 78

peculiarity of . . . . vii 77

percussion in . . . . vii 79

prognosis of . . . . vii 81

rapid progress of . . . vii 79

respiration in . . . . vii 78

rhonchi in . . . . vii 80

symptoms of . . . . vii 78

the pulse in . . . . vii 81

remittent fever, cause of. . xii 95

therapeutics, &c. report on xx 551, xxiii 299

Infant hospitals in Paris, deaths in xii 350

life, effect of malaria on . . xviii 511

mortality in Wirtemberg . vii 186

schools, remarks on . . i 342

Infant, animal heat in relation to . x 520

bed-clothing of . . . . x 522

constitution of at birth . . x 519

cranium of, peculiarities of . vii 89

crying of, remarks on . . x 523

crying in utero, case of . . i 607

dead, signs of its term of life . ii 406

exercise of . . . . . x 526

food of, at birth . . . x 522

how often to be suckled . . x 523

human, reflex action in . . xxii 509

indentation of os frontis in . xiv 236

liability of, to pneumonia . x 521

lungs of, artificial inflation of . vi 64

marks on its neck by the uterus ii 416

newborn, ecchymosis in . . vii 90

on proofs of life in the . ii 408

nursery and clothing of . . x 521

on bathing the . . . . x 525

on cleansing the . . . x 525

pure air important to . . x 520

rapidity of circulation in . x 520

reflex actions, pure, in . . xxii 509

remarks on weaning of . . x 524

resuscitation of a newborn . v 512

revival of an, after burial . v 582

sleep and sleeplessness of . x 526-7

unnecessary medicine to . x 525

Infants, abuse of calomel to . . iii 442

anatomical characters of . ii 406

apoplexy of newborn . . vii 92

remarks on . . . ii 360

artificial food to, thrush from . xxiv 431

asphyxia in, treatment of . v 277

at the breast, pulse of . . xiii 547

best time of examining . . vii 75

bloody cranial tumours of . vii 86

bloody tumours of scalp in . i 182

blue diseases of . . . . xi 208

cause of easy vomiting in . ii 537, 540

cerebro-spinal diseases of . xi 208

clinical observation of . . vii 75

colour of skin in . . . xi 199

conduct of their temper . . ii 222

convulsions of . . . . xx 363

cutaneous diseases of . . vii 93

daily scarification of gums of . xxiii 197

deep yellow diarrhea of . . iii 444

diarrhea of, observations on . xx 359

treatment of . . . xxiv 47

disease of circulation of . . xi 208

diseases of, auscultation in . vii 77

newborn . . . vii 74

double pneumonia in . . vii 78, 80

Dr. Billard on diseases of . xi 198, 209

Dr. Maunsell on sleep of . iii 442

effects of feeding, with arrow-root xxiii 498

entero-colitis of . . . . xx 360

erysipelas in, remarks on . xxii 548

Infants, evils of artificially feeding . vii 569

examination of the pulse of . vii 76

exfoliation of epidermis in . xi 200

exposure of, to the open air . iv 305

facial contractions in . . vii 75

food of, remarks on . . ii 222

gangrene of mouth of . iii 444, xi 203

gin-sling and milk-punch to . v 412

great secretion of mucus in . xi 205

heat of . . . . . xii 145

hospital for, in Paris . . xi 198

hydr. c. creta to, advised . iii 442

improper mode of feeding . ii 385

induration of cellular tissue in ii 361, xi 202

analysis of ii 363

pathology of ii 363

intestinal villi in . . . . i 525

Jadelot on diseases of . . vii 75

lungs of, floating of . . ii 412

means of expression in . . xi 201

measurement of heads of . x 244

M. Ricord's plan of quieting . vii 77

myelitis of . . . . . xiii 248

newborn, cerebral hemorrhage in vii 92

diseases of . . . . xxi 467

pemphigus in . . . vii 94

pustules in . . . . vii 93

strictures on food to . ii 383

weight of lungs of . . . ii 410

normal frequency of pulse in . vii 76

on marks of contusion in dead ii 415

on shedding of tears by . . xi 201

peculiarities of . . . . iii 440

physiognomical semeiology of xi 201

pleurisy in . . . . . vii 82

pneumonia in . i 253, vii 77, xx 362

peculiar . . . . . xi 207

site of . . . . . vii 78

poisoning of, by opiates xviii 504, 558

posseting of . . . . xx 238

pulse of . . . . . xix 390

with entire funis . . vii 76

purulent ophthalmia of iii 443, xviii 410

Rau on mortality of . . vii 591

registration of, in France . i 333

relative mortality of . . ix 350

remark on the thirst of . . v 413

separation of funis in . . xi 200

state of respiration in . . xx 358

stillborn, cold water to . . xii 560

weight of lungs of . . ii 411

strangulation of, by the funis . ii 415

tables of the pulses of . . v 268

tears of, Billard on . . ii 356

the cerebro-spinal system of . iii 440

the face in diseases of . . vii 75

the mean heat of at birth . iii 440

their sufferings from nurses . ix 532

to be soon put to the breast . iii 441

treatment of diarrhea of . iii 444

treatise on diseases of . . xx 357

trismus in newborn . . x 275

unnatural management of . ii 385

Valleix's plan of composing . vii 77

Infants, vapour-baths unsafe for . xi 209

various lengths of intestines in ii 363

washing and clothing of, remarks on ii 222

weaning of, remarks on . . ii 386

Infection, and contagion, distinction of xv 381

caused by animalcules . . ix 398

from dead bodies, remarks on i 378

purulent, theory of . . xx 218

Infiltration, Dr. Walshe on . . xxii 33

insular tubercular . . . xxiii 431

Infinitesimal doses in homœopathy xxi 228

Infirmaries in Ireland, defects of . ii 186-7

history of . . ii 186

Infirmary accommodation in Ireland ii 185

Infirmary, Edinburgh, report from . v 601

Glasgow, report of . . . v 607

Inflamed parts, changes in, after death iv 138

Inflammation, abdominal . . xxiii 126

diagnosis of . xii 280

action of . . . . . xx 297

adhesive, Dr. Macartney on . vii 431

Mr. Mayo on . . . iii 61

of liver . . . . . xxi 49

remarks on . . . iii 62

Sir A Cooper on . . vii 431

affection of system in . . xviii 71

after section or disease of nerves xvii 582

anatomical characters of . viii 193

and fever, distinction of . i 30

and ulceration of os and cervix uteri . . . . . . xxiii 293

appearances resembling . . viii 195

asthenic theory of . xviii 112, 114

attraction-theory of . . xvii 577

best studied in frogs . . iii 213

blood and vessels in, *figure of* xviii 108

bloodletting in . . . xviii 72

buffy coat of blood in . . xvii 147

cause of intense pain in . . iii 24

cerebral, emetic tartar in . i 421

changes in blood in viii 190, 192, xviii 255

in tissues from . . viii 193

produced by . . viii 190

chronic, colours of . . . viii 194

circulation in . . . viii 190

cold applications in . . v 462

by irrigation in . . viii 200

colourless corpuscles in . . xvii 569

compound globules in . . x 259

constitutional . . . i 252, 254

Mr. Travers on . . ii 22

constriction in . . xvii 571, 579

consulting feelings in . . viii 200

cutaneous, seats of . . ii 148

deaths from, in England . ix 356

deceptive nature of . . iv 185

definitions of . . . iv 34

development of . . . iii 214

differences of . . . viii 516

diffuse . . . . . ix 578

doctrine of, in France . . iii 448

Dr. Alison's work on . . x 235

Dr. Bene on . . . . i 8

Dr. Bennet on . . . xix 244

Inflammation, Dr. Billing's view of . . v 201

Dr. Canstatt on . . xiii 334, 336

Dr. Carswell on . . . vii 417

his view of . . . viii 189

Dr. Graves on . . . xvi 257

Dr. Gross on . . . xxiii 67

Dr. Henle's theory of . . ix 409

Dr. Klencke on . . . xvi 503

Dr. Lanza on . . . xxiii 55

Dr. Lebert on . . . xxii 19

Dr. M. Payne on . . . xi 398

Dr. Macartney on . . vii 221, 417, 426

his theory of . . viii 184, 187

Dr. Stevens's pathology of . xiii 203

Dr. Watson on mercury in . xvii 125

Dr. Williams on . . xviii 91, 106

effect of civilization on . . xviii 251

effusions from . . . viii 191

electrical apparatus for . . viii 409

cure of . . . viii 408, 409

nature of . . . viii 408

excess of fibrin in . . . xviii 257

exudation of fibrin in . . xviii 72

exciting cause of . . . xvii 581

exudation in . . . . xvii 582

exuded matter in . . . xviii 271

fanaticism, remarks on . . xvii 107

first steps of . . . . xvii 568

fomentations in . . . viii 201

former treatment of . . i 398

from the blood . . . xiii 335

from the nerves . . . xiii 335

gangrenous, characteristics of . ii 28

diagnosis of . . . . ii 23

fever in . . . . . ii 28

Mr. Travers on . . . ii 28

pulse in . . . . . ii 29

treatment of . . . . ii 30

general bleeding in . . viii 198

general remarks on . . viii 169

heat in . . . . . . xiii 335

Hunter, John, on . . vii 417, 420

his ideas of . . . viii 183

principle of . . vi 112

in aged people . . . xvii 107

increased vital action in . . viii 190

increase of fibrin in . xviii 102, 105

in dysentery . . . . xxiv 338

increase of bulk from . . viii 197

in relation to nerves . . iii 14, 15

in reparation of parts . . x 463

in vegetables . . . vii 427, 429

intention by bleeding in . . viii 198

its influence in practice . . iii 447

late chemical views on . . xviii 114

local, on bloodletting in . . xviii 72

Magendie's views of . . viii 339

medical, in France . . . iii 448

mercury in . x 532, xiii 338, xix 527

misconceptions on . . . ii 16

modified treatment of . . x 534

Mr. Addison on . . xviii 91, xx 121

Mr. Jones on the blood in . xviii 255

Inflammation, Mr. Macilwain's law of vi 112

Mr. Mayo on the nature of . iii 59

Mr. Morgan on . iv 185, 186, x 532

Mr. Travers on . . . xviii 69

Mr. W. Jones's report on . xvii 567

nature of, remarks on . . xx 147

nauseating medicines in . . viii 199

nervous influence in . . xvii 579

no immediate cure of . . i 398

none, in worms, &c. . . vii 427

observations on . . . xii 127

of bladder, Mr. Coulson on . vi 510

of cornea, nature of . . xvii 585

of eye, Van der Kolk on . . xviii 48

of lymphatics, M. Velpeau on ii 556

of muscles, case of . . . iii 227

office of the nerves in . . viii 188

of nervous centres . . . xvii 528

of non-vascular parts . . xvii 585

of uterus, acute . . . xvii 506

of vermiform process . . vii 551

opposite conditions in . . viii 191

order of parts liable to . . xiii 335

our knowledge of . . . viii 512

oxalic acid in . . . xii 539

pain and sensibility in . . viii 198

pathology of . . . . viii 513

phenomena of . . . iii 215

explained . . . viii 189

plasticity of blood in . . xx 71

primary affection in . viii 189, 191

Professor Naumann on . . iii 213

proximate cause of . i 11, viii 187

Rasori's practice in . . viii 204

his views on . . . viii 203

red corpuscles in . . . xvii 569

reduction of heat in . . viii 200

relaxation in . . . . xvii 571

remedies for . . . . viii 198

resolution of . . . xviii 279

retardation of blood in . . xvii 568

Schönlein on . . . . xxi 22

secondary, symptoms of . . iii 505

Mr. Travers on . . ii 13

sensibility in . . . . viii 189

signs of, after death . viii 196, 203

simple and specific . . ii 19

specific, Mr. Travers on . . ii 18

stagnation of blood in iii 59, xvii 568, 576

state of capillaries in . . xvi 257

the plasma in . . . xvii 570

the vessels in . . viii 183, 186

sthenic, effects of . . . xviii 103

summary remarks on . . xviii 113

surgical, in England . . iii 448

terminations of . . . xviii 268

the blood-corpuscles in . . xvii 93

theory of, by Rasori . vii 417, 424

the white corpuscles in . . xviii 105

thoracic, pathology of . . xx 79

topical bleeding in . . . viii 199

treatises on . . . . viii 169

treatment of . vii 442, x 463, xiii 336

Inflammation, vascular dilatation in xvii 571, 579

Inflammations, changes of the blood in xvii 144

on intermittent . . . viii 421

specific, pathology of . . iii 18

tubercular, M. Rayer on . ii 155

visceral, left to nature . . xxii 559

Inflammatory affections of the brain xxii 384

and neuralgic pains . . iii 25

blood, disquisition on . . iii 67

Mr. Mayo on . . . iii 66

diathesis, theory on . . xvi 227

" couleur ardoisée" . . viii 194

diseases, effect of season on xxiv 110, 113

fever, case of . . . . xv 409

globule, compound . . xxii 333

process, results of . . . xviii 110

two periods of . . xiii 334

products of tissues . . . xxiv 118

redness after death . . viii 195

forms of . . . . viii 193

uterine affections . . . xviii 523

Inflating infants' lungs, remark on . viii 474

the lungs, on the mode of . xxii 163

Inflation, artificial, of the lungs . xvi 310

of the lungs, death from . vii 519

Influenza as to sex and age . . vii 97

atmosphere after . . . xii 111

bibliography of . . . vii 104

bloodletting in . . . vii 101

cause of . . . . . v 557

characteristics of . . . vii 108

contagiousness of . iii 455, vii 99

cosmic phenomena with . . vii 104

course of the epidemic . . vii 104

death of the old in, cause of . vii 113

deaths from . . . . xii 111

on depletive measures in . xvi 244

descriptions of . . . vii 106-7

diet in . . . . . . vii 102

Dr. Baird's practice in . . vii 102

Dr. Craigie's account of . . iii 455

Dr. Graves on . . . xvi 243

duration of . . . . vii 98

early existence of . . . vii 103

electric hypothesis of . . vii 113

epidemic, account of . . xx 231

epidemics of, list of . . xx 445

false membrane in lungs in . vii 103

four stages of . . . vii 105

history of . . . . . xx 444

in Birmingham, 1837 . . v 214

Copenhagen, 1836-7 . . v 558

horses . . . . . xxiv 85

Lisbon, 1837 . . . v 557

relation to weather . . vii 103

intermittence in . . . viii 503

like other epidemics . . vii 113

the exanthemata . . vii 113

Mr. Myles's practice in . . vii 102

of 1510 . . . . vii 108

1830-2 . . . . vii 105

1834 . . . . vii 105

1837, history of . . . vii 95

Influenza, symptoms of . . . vii 99

treatment of . . . vii 101

open air in relation to . . vii 98

proclivity to relapse in . . xx 447

proportion of deaths in . . vii 98

proximate cause of . . vii 112

rapid respiration in . . xii 111

relapses in . . . . . vii 98

season of its appearance . vii 96

source of danger in . . xii 111

some remarks on . . . viii 332

spasmodic character of . . vii 113

terms applied to . . . vii 105

three principal forms of . . vii 106

transmission of . . . v 557

treatment of . . . . xx 446

use of opium in . . . vii 102

why not contagious . . iii 455

Influenzas of 14th and 15th centuries vii 108

of 16th and 17th centuries . vii 108

18th century . . . vii 109-112

late years, remarks on . viii 503

the earlier, treatment in . . vii 112

Influenzoid affections . . xii 111

Infusoria, circulation in the . vii 181, xii 245

in pus-corpuscles . . . xxii 332

in blood, experiment on . . xxii 336

in serum of blood . . . viii 351

production of . . xix 169, 180

Infusory animals, production of . i 392

Ingesta, due proportion of liquid in xvii 41

rate of assimilation of . . x 101

Ingestion and egestion, Dr. Hall on xiv 197

Inglis, Dr., on bronchocele . . viii 103

on the skull of Eugene Aram . vi 581

Ingleby, Mr., his cases in midwifery iv 398

Inguinal glands, in fungous testicle i 473

hernia, direct, operation for . xxii 205

Inhalent apparatus of skin . . ii 440

Inhalation of flame . . . v 439, 440

of iodine and conium . . v 606

Inhalations, observations on . . xvi 204

on the benefit from . . xix 15

Inhaling powders, mode of . . xviii 296

Injection of tincture of iodine, Velpeau on . . . xviii 88, 90

Injections, Mr. Mayo on the use of v 392

use of, in hydarthrosis, &c. . xxii 71

uterine, experiments on . . xii 263

vaginal and uterine . . xii 216

Injuries, complication of, fatal . v 175

local, organic disease after . ii 14

on death by many small . iii 251

Injury, death from slight local . ix 61

Innate ideas, the doctrine of . . v 82, 83

Innervation, suspended, effects of . viii 332

Innis, Capt., remarkable case of . vii 60

Innkeepers, liability of, to dropsy . vi 19

Innominata, aneurism of . . xxiv 420

Inoculated smallpox, not contagious xxi 361

Inoculation, abolition of variolous . x 300

by contagious cells . . xx 214

experiments on . . . xx 406

Inoculation of Catherine II. of Russia . i 605

cholera . . . . . ix 245

meaning of the term . . i 375

of measles, results of . . xv 235

morphia, &c. . . . . iv 506

smallpox, history of . . vi 484

remarks on . . . xii 97

syphilis, experiments on . x 382, 387

with ova of hydatids . . xx 413

tænia solium . . . . xx 418

Inosinic acid, Liebig on . . xxiv 267

Insane epileptics, management of . ix 157

foxhunters, employment of . ix 171

gentleman, his narrative . xi 104

patients, liberation of . . xix 340

wards in prisons recommended xxiv 236

Insane, the, on acute delirium in . xxi 418

amusements for . . xiii 415

amusements of, in America . v 239

arguments for severity to . xi 123

asylum for, at Berlin, bad . i 500

asylums for, mortality in xxii 350, 360

attendants on . ix 163, xiii 283, 414

basis of classification of . ix 153

benefit of asylums to . . xi 124

boys as attendants on . . xx 480

Brompton establishment for . iii 189

claims of . . . . ix 186

classification of . . . xix 332

coercion-chairs for . . . ix 159

competency of evidence by . x 157, 174

confinement of, by straps . ix 157

conscientiousness, case of . vii 12

contrivances in Hanwell for . xix 598

crimes committed by . . xv 89

Danish hospital for . . iii 568

depravity in . . . . xii 54, 60

destruction of clothes by . ix 159

Dr. Allen on . . . . vii 1, 46

Dr. Conolly on treatment of . xiii 274

dress for the destructive . ix 159

effects of idleness on . . xi 113

entertainments of, at Hanwell. xv 221

establishments for . . . ix 144

evils of restraint of . . ix 156

farm labour by . . xix 295, 333

feeding of . . . . ix 168

flogging . . . . ix 149

forcible restraint of . xiii 416, 419

former cruel treatment of . vii 3, 4

severity to . . . xix 296

treatment of the . xiii 413, 416

French asylums for the . . ix 152

frequency of phthisis in . . v 375

of pulse in . . . iii 224

gangrene of the lungs in . ii 546

general treatment of . . xiii 283

ground-floor best for . ix 151, 153

hospital for, at Rome, bad . i 498

for, at Sonnenstein . . i 499

importance of cleanliness to . xxiv 159

of the charge of the . ix 188

injudicious language to the . xi 111

Insane, the, injustice to, still possible vii 3

instinct of ferocity in . . xx 28

instruction of, at Hanwell . xv 222

irresponsibility of . . . xvi 87, 273

knives and forks to . . ix 168

labour by . . . . ix 170

labours of, in America . . v 240

law on confinement of the . x 174

long fasting of the . . . ii 547, 548

management of, at Hanwell . xi 127

medical treatment of . . xix 330

mischief of confining . . xi 109

mode of opening the mouth of ix 168

moral qualities of . . . xii 60

management of . . i 286

in America v 239

treatment of . . . viii 584

motives to crime in . . xvi 90

M. Leuret on the douche for . xi 124

Mr. Tuke on restraint of . xiii 414

neuralgic pains in . . . iv 133

occupation of . . xix 295, 333

of the middle classes, asylum for xx 479

on benevolent attention to . xxiv 165

embarrassed pronunciation in iii 226

gaining the confidence of . xi 126

murder by . . . . x 134

reasoning with . . . xxi 463

vacillating walking in . . iii 226

progress of non-restraint of . xi 116

propensity to suicide in . xi 120

public worship by . . xii 55

qualifications of attendants on . xi 123

physician for . . . xix 601

refusal of food by . . ii 547, ix 168

religious exercises of . v 242, xxi 464

instruction to . . . xii 54

services to . xiii 284, 416, xix 339

restraint of . . . . xix 334-9

remarks on . . xix 283, 297

Retreat at York for . ix 144, 153

seclusion of . . . . xiii 413

separate buildings for . . ix 152

hospitals for . . . ix 150

signs in, indicating tonics . iii 223-4

social meetings of . . . v 241

starvation of, effects of . . ii 547

sympathy with . . . vii 42

table of occupations of . . vii 52

temperature of . . . xii 146

temporary restraint of . ix 155, 158

their ideas of themselves . x 132

the physician qualified for . ix 150

treatment of, at Hanwell . xv 25

Dr. Seymour on . . xxiv 153

in Denmark . . . iii 571

in Pennsylvania . . viii 583

use of music to . . . xiii 416

violence, causes of, illusory . x 154

wills made by . . . x 173

Insanity, absence of motive in . xvi 84

abuse of opium a cause of . vii 29

among religious sects . . vii 28

Insanity, and criminal responsibility xxiv 226

dreaming, identity of . xxiv 191

eccentricity, diagnosis of . x 133

temper, diagnosis of . x 133

apoplexy in . . . vii 30

appearances of the brain in . vii 32-3

at Charenton, tables of . . i 273

atrophy of the brain in . . ii 461

attention to dress in . . xxiv 162

benefit of shower-bath in . xi 127

blisters in . . . . vii 37

bloodletting in iii 225, vii 36, xi 121, xv 467, xxii 56

bodily restraint in . . xiii 274, 282

cardiac disease in . . . xxii 425

case of feigned . . . iii 533

homicidal . . . ix 164

sudden . . . x 134

cases of, by Dr. Jacobi xxii 3, 5, 7, 9

treated by tonics . . iii 225

caused by over-education . iv 61

cause of local affections in . viii 332

causes of, in France . . vii 28

its prevalence . . . iv 61

cessation of phthisis in . . vii 18

clothing and bed in . . vii 37

confounding vice with . . xvi 100

contagiousness of . . . ix 151

convalescents from . . vii 45

counter-irritants in . . vii 38

crises in . . . . . vii 31

curability of . . . . vii 18

cured by injuries of head . vii 28

moral impressions . vii 31

cures in the York Retreat . vii 20

cures of, in various asylums . vii 19

death from restraint in . . xx 478

decline of eruptions in . . vii 18

depletion in . . . . vii 35

diagnosis of a lucid interval in x 134, 171

diet and employment in . . xxiv 162

difficult legal cases of . . x 131

difficulties in relation to . . vii 2

discordant pulsation in . . xxii 13

disease from fasting in, case of ii 547

diseases accompanying . . vii 30

disordered primæ viæ in . vii 18

disputed causes of . . . vii 34

Dr. Allen's theory of . . vii 47

Dr. Conolly on restraint in . xi 126

on treatment of . . xv 220

Dr. Herzog on . . . xxi 460

Dr. Hull on . . . xvii 203

Dr. Jacobi on the forms of . xxi 1

Dr. Prichard on . . . vii 1, 5

his division of . . vii 5

on delusion in . . xvi 82

on moral . . . xvi 82

Dr. Steward's classification of xxii 59

Dr. Thomas Mayo on . . xxiv 225

Dr. Thurnam's statistics of . xxii 349

drinking as a cause of . . vii 26, 29

duration of, in asylums . . xxii 354

Insanity, education in relation to . vii 25

effect of civilization on . . xviii 239

enteric disorder a cause of . vii 29, 30

epidemic character of . . vii 29

errors of diet a cause of . . vii 26

exercise in open air in . . xxiv 160

family, important fact on . vii 59

fasting in, hurtful to mind . ii 548

treatment of . . . ii 548

febrile symptoms in acute . vii 18

feigned, diagnosis of . . x 136

French law of interdict in . x 173

frequency of, among Quakers . xv 55

from normal disorder . . xx 13

moral feelings . . xxiii 236

undue lactation . . xii 329

wounds of the head . xxi 464

fewer cases of, among Quakers vii 25

general paralysis in . vii 14, xx 29

treatment of . . . vii 40

hallucinations in . . . x 166

Hanwell dose for . . . vii 39

heart and arteries in acute . vii 18

hereditary nature of . . xiii 259

homicidal . . xvi 83, 91, 109

principle of . . . x 157

Mr. Taylor on . . xxi 486

illusions in . . . x 166

impaired volition in . . x 166

importance of seclusion in . xi 127

improved treatment of . . vii 3

in agricultural life . . . vii 51

inappetence in, sketch of . vii 10

in Bethlem hospital . . xviii 371

in conduct, not in ideas . . vii 8

increased strength in . . xxii 15

in crimes, question of . . xxiv 241

incision of scalp in . . vii 38

in epileptics . . . x 171

in families . . . vii 22, 23

misery from . . . ix 187

inflammatory nature of . . vii 34

in mountainous districts . . vii 51

Norway, tabular views of . i 278

prisons, Dr. Holst on . . xii 502

prostitutes in Paris . . iv 70

relation to age . . . vii 24

climate . . . vii 28, 37

marriage . . . vii 27

menstruation . . xiv 395

phrenology . . xx 34

season . . . vii 29

the sexes . . vii 23, 51

in Saxony, statistics of . . vi 240

intermittent, case of . . i 608

in the agricultural poor . . v 69

interdiction and restraint in . x 161

intermittence of . . . vii 30

intestinal canal in . . . vii 29

in the honest labourer . . ix 187

rich and poor . . ix 187

in Westphalia, statistics of . vi 240

issues and setons in . . vii 38

| | | |
|---|---|---|
| Insanity, Jacobi's classification of | xxii | 2 |
| peculiar views of | xxii | 17 |
| jurisprudence of, treatises on | x | 129 |
| law-definitions of | x | 130 |
| legal relations of | xix | 33 |
| liability to, at different ages | xxii | 361 |
| in Quakers | xxii | 362 |
| in the sexes | xxii | 360 |
| local bleedings in | vii | 36 |
| lucid intervals in | x | 171, 173 |
| magnetising in | vii | 40 |
| maniacal, rarely fatal | vii | 33 |
| man's self-control of | xvii | 245 |
| manustupration as a cause of | vii | 29, 54 |
| medical certificates of | x | 163 |
| competency as to | x | 165 |
| evidence in x 155, xvi 94, 100, 110, 274 | | |
| jurisprudence of | x | 129 |
| witnesses in | x | 168 |
| treatment of | xv | 468 |
| mental pathology of | xxiii | 226 |
| mercury, as a cause of | vii | 29 |
| mode of confinement in | vii | 44 |
| moral, and intellectual | vii | 5, 6 |
| causes of | vii 25, 28, xi | 120 |
| most common | vii | 25, 28 |
| remarks on | vii | 6 |
| treatment of | vii 42, xxii | 60 |
| morbid anatomy of head in | vii | 31 |
| mortality, as to age in | vii | 22 |
| in, Esquirol on | vii | 21 |
| M. Esquirol's definitions of | vii | 5 |
| Mr. Browne on statistics of | v | 68 |
| Mr. Farr's work on | vii | 19 |
| Mr. Neville on | iii | 189 |
| Mr. Newnham on | xv | 421 |
| Mr. Tuke's table of cures of | vii | 20 |
| nauseating doses in | vii | 39 |
| necessity of restraint in | x | 133 |
| new views of, by Dr. Wigan | xx | 11 |
| obesity and emaciation in | vii | 31 |
| observations on | xxii | 384 |
| of many years, cured | vii | 19 |
| of same form in families | vii | 23 |
| oftenest from female parent | vii | 23 |
| of the intellectual faculties | x | 132 |
| of the moral faculties | x | 132 |
| of the moral sentiments | xv | 468 |
| on all crime arising from | x | 154 |
| opposition to medicine in | vii | 38 |
| paralysis in, treatment of | vii | 41 |
| partial | xvi | 82-3 |
| irresponsibility of | x | 154 |
| pathological causes of | vii | 33 |
| period of incubation of | xx | 19 |
| phrenological views of | v | 67 |
| physical causes of | vii | 28, 33-4 |
| plea of, in criminal cases | xvi | 81, 273 |
| the twelve judges on | xvi | 273 |
| precept and maxim in treatment of | xi | 126 |
| predisposing causes of | vii 22, ix | 187 |
| prevalence of, in America | iv | 59, 61 |
| prevalent in free countries | iv | 61 |
| Insanity, preventing injury in | vii | 43 |
| prognosis in | vii | 18 |
| proportion of cures of | vii | 19 |
| proximate causes of | xvii | 526 |
| public events, in relation to | vii | 26 |
| puerperal, paleness of brain in | vii | 32 |
| purgatives in | vii | 38 |
| recovery from | vii | 18 |
| reflections on | ix | 186 |
| refusal of food in | vii | 44 |
| relapses of | xxii | 351 |
| religion in relation to | xxiv | 164 |
| religious, case of | i | 287 |
| Mr. Newnham on | xv | 422, 429 |
| remark of Dr. Holland on | viii | 488 |
| on medicines in | vii | 40 |
| remarks on cases of | vii | 2 |
| on the causes of | i | 275 |
| remissions in | x | 171 |
| remittence of | vii | 31 |
| responsibility for crime in | x | 139, 166 |
| restraint in, on pleas for | xix | 607 |
| results of, in relation to age | xxii | 352 |
| in relation to sex | xxii | 350 |
| rotatory motion in | vii | 40 |
| seclusion in | xiii | 279 |
| shape of external ear in | xix | 596 |
| shaving the head in | vii | 37 |
| Sir Alex. Crichton on | xv | 467 |
| Sir W. Ellis on | vii | 1, 47 |
| sleep in, Dr. Conolly on | xxiv | 152 |
| sooner or later, from epilepsy | x | 171 |
| statistics of | xix | 341 |
| at Charenton | i | 272 |
| strait-waistcoat in | vii | 43 |
| suspension of discharges in | vii | 18 |
| table of causes of | vii | 9 |
| tables of cures in periods of | vii | 21, 51 |
| temporary | x | 171 |
| test of responsibility in | x | 139, 153 |
| the memory in | x | 132 |
| the pulse in | xxii | 12 |
| therapeutics in | xi | 121 |
| treatises on | vii 1, 2, xi | 104 |
| treatment of | vii | 34 |
| first period of | vii | 43 |
| unimproved English law of | x | 153 |
| unsettled medical practice in | vii | 3 |
| use of acetate of morphia in | ii | 167 |
| aconite in | xi | 121 |
| camphor in | vii | 39 |
| digitalis in | vii | 39 |
| douche in | vii 37, xi | 125 |
| emetics in | vii | 38 |
| hyoscyamus in | vii | 39 |
| mercury in | vii | 40 |
| narcotics in | xxii | 55 |
| opium in | vii | 39 |
| and narcotics in | xi | 121 |
| sea-voyages in | xx | 39 |
| tonics in | iii | 223 |
| warm-baths in | xxii | 58 |
| vagueness of theories of | vii | 3 |

Insanity, various treatises on . . vii 1

visceral diseases in . . . vii 33

voices and spirits in . . xi 107

warm and cold baths in . . vii 37

with congestion of brain . . iii 224

Insect, eggs of, long latent vitality of iv 130

larvæ in the alimentary canal . xii 219

life, as a cause of disease . . viii 506

Dr. Badham on . . . xxi 494

-propagation of cholera . . xxiii 334

Insects, aboriginal creation of . . xix 171

absence of sensation in . . xxi 494

and worms, used as food . . xvii 16

camphor poisonous to . . iv 130

circulation of the blood in . ii 213

J. Hunter's preparations of . xv 215

larvæ of, passed by stool . . xiii 540

locomotive power of . . xi 97

Mr. Newport on . . . ix 533

nervous system of . . . xvii 187

in, Newport on . xx 487

none universally diffused . . iii 369

of different species, in copula . xxiv 440

respiration of, Mr. Newport on iii 292

restraint of hemorrhage in . xx 506

sensibility of . . . . v 545

spinal cord of . . . v 500

temperature of . . iv 527, xii 144

tracheæ, or air-tubes of . . vii 170

wonderful instincts of . . xi 98

Inquests, coroners', remarks on . ix 53

on sudden deaths, remarks on . xv 82

Insolation, in rheumatism . . xiv 420

on cerebral affection from . iii 305, 310

Insomnia, in anemia, cold foot-baths in xxii 54

Insomnium, in insanity, treatment of xxii 56

Inspection, ocular, of chest, its use . i 480

Inspections, on medico-legal . . ix 50

Inspiration, full, unknown to some . v 145

in relation to the pulse . . iv 242

muscles of, Stromeyer on . v 133

physical characters of . . ix 308

Inspiratory murmur, cessation of . ix 309

muscles, functions of the . . v 134

sound, abnormal . . . ix 308

changed qualities of . ix 309

Instinct, and instinctive actions v 491, 505, 517

and reason, Dr. Bushnan on . xi 90, 103

essay on . . xvii 525

application of the term . . xi 90

Dr. Alison on . . . xi 90

infant guided by pure . . xi 100

in the sick . . . . i 191

nature and modifications of . xxiv 192

remarkable, of insects . . xi 98

the will, in relation to . . xi 90

Instincts, in man, remarks on . . xi 100

lesions or perversions of . . xx 25

mental . . . . . . xi 101

of animals, Mr. Swainson on . xi 90, 103

sensational, Unzer on . . xxiv 199

vital, in plants and animals . iv 30

Instinctive actions, characteristics of xi 96

Instinctive actions, classification of . xi 99

Dr. Carpenter on . iv 530

in man . . . v 517

localization of the . xiii 482

object of . . . xi 102

partly voluntary . v 506

remarks on . . v 106

Sir G. Blane on . v 529

uniformity of . . v 517

acts, theory of . . . xix 306

movements, nature of . . xxii 505

reflex, and emotional actions, analogy between . . . xxiv 207

Institution de Sainte Perine, account of i 281

Institutions, medical foreign . . i 289

in America . . iii 273

of United States . ii 583, 593

Instrumental labours, in Guy's Hospital iv 372

Instruments, surgical, gilding of . xiv 233

Insufflation of lungs, in infanticide . ii 413

Insurance companies, and doctors . xiii 578

offices, (see Assurance.)

tables of the Equitable Society iii 40

Intelligence, low, in animals . . xi 102

pure, kingdom of . . . xi 102

Intellect, on delusions of . . xx 13

overtasking, remarks on . . iv 56

sharpening of, in old age . vi 80

Intellectual education . . . ix 412

powers, varieties in the . . xi 95

young women, rarity of . . v 408

Intemperance, and mortality, in Glasgow xxiv 518

deaths from, in Ireland . . xviii 41, 44

dropsy in relation to . . vi 18

in drinking, remarks on . . ii 388

in Ireland, history of . . xviii 41

injurious effects of . . . xx 423, 427

the great cause of crime . . xxiv 517

Intercellular substance of plants . ix 502

Intercostal artery, death from wound of iii 536

muscles, action of the . . v 139

paralysis of . . . v 424

spaces in the sexes . . . xvii 254

Intercostals, in respiration . . xvii 255

Interdicts, French law on . . x 173

Intermarriage, Mr. Walker on . vii 370, 385

on rational principles . . vii 384

Interment, delay of, evils from . xvii 404

in towns, committee on . . xv 513

Dr. Reid on . . . xix 4

Mr. Chadwick on . . xv 524

report on . . xv 513, xvii 403

Mr. Walker on . . . xv 542

Interments, French law on . . x 212

on classifying . . . xv 522

Intermittence, definition of Cowan's iii 163

Dr. Cowan on . . . iii 162

in health and disease . . iii 163

in nervous system . . . iii 163

nature of . . . . viii 505

Intermittent fever, algide . . viii 62, 66

and phthisis, relation of xx 105

Intermittent fever, at Bona . . viii 59
chloride of soda in ii 553
comatose . . viii 62
delirious . . viii 62
Dr. Kremers on . viii 57
his theory of viii 67
Dr. Maillot on . viii 56
etiology of . . iii 105
experiments on . iii 106
M. Brachet on . iii 105
narcotine in . viii 263
pain of back in . x 557
pernicious . . viii 61
relapses of . . viii 65
remedies for . viii 64
spinal pain in . viii 67
state of spleen in xii 93, xx 237
theory of . . viii 60
treated by salicine i 571
treatises on . . viii 56
treatment of ii 176, xx 238
tying limbs in . viii 251
various forms of . viii 61-3
(see Ague.)
Intermittent fevers, Dr. Leitao on . v 558
inflammations . . . . viii 421
Intermittents, bleeding in the cold stage iii 104-5
on the cold stage of . . iii 104
in Bengal, treatment of . . iii 348
bloodletting in . . . viii 64, 66
effects of temperature on . viii 61
homœopathy in . . . xxiii 608
on periodicity in . . . xviii 235
on quinine in . viii 61, 64, 65, 66
use of cetrarin in . . . . xiii 67
Internuncial function of nervous system v 490
Interparietal bone in Peruvian skulls xviii 384
Interrupted suture of intestines xxiii 19
Interstitial pregnancy, case of . xxiii 278
Intertropical disease, cause of . xiii 351
Intestinal calculi, L'Heritier on . xvii 446
canal, closure of, by tumour . xiv 231
congenital closure of . xiii 508
diseases of . . . xviii 310
diverticula of . . . xiv 360
moulting of . . . xvi 223
of infants, lengths of . ii 363
on the gases of . . xvii 436
tubercles in . . . xxiii 432
capillaries, Dr. Gaddi on . xii 529
diseases, prevalence of . vi 26
fever, sources of (App. to vol.) xi 35
fistula, from hernia . . xxii 202
follicles, ulceration of . . xv 95, 97
glands, anatomy of . . vi 218
Dr. Boehm on . . i 236
*plate of* . . . . i 528
structure of . . . i 236, 521
invagination in the cow . . xii 253
mucous membrane, inflammation of xv 94
obstructions, diagnosis in . xxiii 419
treatment of . . . xxiii 420
secretions, L'Heritier on . . xvii 434
Intestinal strangulation in a mare . xii 538
internal . iii 495, iv 168, xii 551
causes of . . iii 496-9
rotatory . . . iii 496
remarks on . . . iii 500
tube, Dr. O'Beirne's improved vi 560
typhous ulcers . . . xv 97
ulcers, cicatrization of . . xvi 433
in dysentery . . . xviii 317
ulceration in typhus . . iii 260
villi in infants . . . i 525
moulting-cells of . . xvi 224
structure of . . . xiv 566
vesicles in cholera . . . i 236
worms, observations on . . xx 416
Intestine, excision of part of, and cure iii 517
great loss of, cases of . . xxiii 26
large, mucous coat of . . xxiv 335
small, adipose tumours in . xxii 474
suture of, case of . . . iv 512
Intestines, action of ligatures on . xxiii 14
cancer of . . . . xxii 299
cases of foreign bodies in . iii 236
changes of, in typhoid fever . ii 48
crystalline deposits in . . xvi 149
dark discoloration of . . i 137
diseases of, in animals . . xxiv 83
effects of ligature on . . iv 512
experiments with sutures of . xxiii 17
hemorrhage from, remark on . vi 139
interrupted suture of . . xxiii 19
lesions of the small . . xi 419
long, in insects not carnivorous i 538
nature of wounds of . . xxiii 2
perforation of, opium in . . ii 61
works on . . . ii 62
peristaltic motions of . . xiii 482
protruded, reduction of . . xxiii 8
relative length of, in insects . i 538
severed, invagination of . . xxiii 21
small, intussusception in . xi 419
strangulated from pressure . iii 495
suture of . . . . . iv 512
wounded, reparation of . . xxiii 15
on sutures . . . xxiii 9, 16
wounds of . . . . xxiii 1
John Bell's method with xxiii 12
without protrusion . . xxiii 6
with protrusion . . xxiii 7
Intestino-vesical fistula, case of . xiv 134
Intoxicating substances, history of xxiii 218
Intoxication, legal responsibility in x 158, 161, 171
tracheotomy in . . . iv 140
validity of deeds in . . x 158
Intra-cranial phlebitis, case of . xx 229
Intra-parietal hernia, after wound xiv 556
Introductory lectures, medical schools' i 203
Introsusception, case of, cured . viii 422
mercurius vivus in . . . viii 422
Intussusceptio cured by inflation . ix 221
intestinal, cases of . . . xxiii 305
Intussusception in infants . . vii 453

Intussusception, mortification from i 146

of small intestines . . . xi 419

treated by inflation . . vi 274

Intuition, considerations on . . vi 447

D'Alembert's definition of . iv 447

mesmeric . . . xix 448, 466

Inunction of mercury, Sir B. Brodie on xxii 169

Invagination, intestinal, in the cow xii 253

of severed intestines . . xxiii 21

of small intestines . . . xi 419

Invalid, Essays by an . . . xviii 472

Invalids, books of travels to . . xviii 475

on companionship to . . xviii 474

female, speaking the truth to . xviii 474

selection of abode for . . xviii 475

self-dissatisfaction in . . xviii 476

temper about trifles in . . xviii 477

temper in . . . . xviii 476

Inventions, repository of useful . xxi 498

Inverness, niceness and naughtiness in xv 334

Inverted uterus and polypus, diagnosis of vi 237

Inversion of uterus . . . iii 425

case of . . vi 236

compression in . viii 55

Dr. Ashwell on . xix 356

Dr. Churchill on . vi 97

late reduction of . vi 97

Lisfranc on . . xviii 22

Mr. Ingleby on . iv 412

Mr. Radford on . v 288

natural cure of . xix 423

report on . xx 530, 542

spontaneous . xxiii 281

the placenta in . xix 357

Inversio uteri, Mr. Crosse's essay on xx 511

Invertebrata, on the heat of . . xii 144

Investigation, on new and old modes of xxiii 154

Investigators, on encouragement to xvii 181

Involuntary acts, expression of . xviii 448

motions, Johnstone on . . i 386

Iodic acid, new method of preparing iii 272

Iodide of iron . . . . . xiii 73

action of . . . v 166

in albuminuria . . xxiv 44

in chlorosis . . xix 346

in syphilides . . xxiii 369

of lead, pills of . . . xiii 364

of mercury, observations on . xiii 74

of potassium as a poison . xx 327

formulæ of . . . xxiii 370

in secondary syphilis . x 415

in syphilides . . . xxiii 369

on the dose of . . xx 486

on large doses of . . xix 518

poisoning from . . x 588

of starch, poisoning from . x 588

of sulphur, use of . . . v 166

Iodides, Dr. A. T. Thomson on poisonous vi 585

of mercury . . . . . v 166

in syphilides . . . xxiii 366

Iodine, action of, on the liver . iii 354

and conium, inhalation of . v 606

and its chemical compounds . v 163

Iodine, angina pectoris cured by . v 165

an insect emitting . . . v 163

-baths for syphilis . . . xix 522

causing absorption of testis . xx 486

caution on prescribing . . iii 354

chronic fluor albus cured by . iii 509

collyrium of . . . . xii 258

Dr. Cogswell on . . . v 162

effects of, in tincture . . v 165

its external use . viii 520

on the skin . . v 164

emaciation produced by . . v 164

exanthema from the use of . xx 518, 523

external use of . . . viii 519

in many diseases viii 521

first medical use of . . v 162

form of tincture of . . . viii 519

in a pure state objectionable . xix 517

in cod-liver oil . . . x 558

in erysipelas . . . . xiv 563

-injection, diseases cured by xviii 89

M. Velpeau on . . xviii 88-90

on gangrene from . . xviii 88

-injections in hydrarthrosis, &c. xxii 71, 78

in ovarian dropsy . . xxiii 296

in serous cysts . . xiii 543

injurious effects of . . . v 164

in mercurial salivation . . iii 511

in opacity of the cornea . . xii 258

in phthisis, new mode of using xii 282

in scrofula, Mr. Phillips on . xxii 152

in the urine of those taking it xxiii 517

its compounds with carbon . v 166

local application of, in syphilis xix 523

medical virtues of . . . v 165

mineral springs containing . xix 518

mode of curing syphilis by xix 516, 522

mode of using externally . viii 520

Mr. Davies on the use of viii 512, 518

occasional bad effects . . viii 120

remarks on its inertness . . v 163

said to produce hepatitis . iii 354

singular effect of, on the urine v 164

the diseases benefited by . v 165

tinct. of, in phagedenic chancre xii 251

injection of, into close cavities xviii 88

treatment of syphilis by . . xx 482

use of, in chronic hepatic disease iii 354

in herpes . . . viii 591

in ovarian cancer . xxii 309

in syphilis . . . v 20

in uterine tumours . iv 373

wide diffusion of, in nature . v 163

Ioduret of iron very easily decomposed ii 210

Ioduretted ointments, singular effect of v 164

solution . . . . . viii 519

waters . . . . xiv 327, 356

Ionian Islands, bowel complaints in ix 68

diseases of lungs in . viii 221

Io pæans of Dr. M. Hall xxiii 193, 195, 197

Ipecacuanha, a special objection to iii 150

emetic for infants . . . xx 361

in chronic dysentery . . iii 145

Ipecacuanha, in dysentery . . iii 331
in erysipelas . . . . xi 327
in typhus fever . . . viii 447
liniment epispastic. . . xvii 554
new preparation of . . vi 247
Iralgia cured by quinine snuff . iii 230
Iranian and Turanian races . . xxiv 454
Ireland, and England, comparative
mortality of . . . xviii 42
causes of death in . . . xviii 35
deaths by murder in, number of xviii 43
from intemperance in . xviii 41, 44
from natural causes in . xviii 44
famine and fever in . . xxiv 285
herb-doctors of . . . xviii 37
intemperance in, history of . xviii 41
medical association of . . x 292
charities of . . . xiii 513
misery and destitution in . xxi 13
mortality in, by consumption . xviii 39
counties in . . xviii 42
from fever . . xviii 39
in ten years . xviii 35
towns in . . xviii 42
number of coroners' inquests in xviii 43, 44
state of the hospitals in . . xiii 515
table of deaths in, by years and ages xviii 43
Iridalgia, Professor Rosa's account of v 34
treatment of . . . . v 34
Irideremia, case of . . . xvi 320
Iridoncus or iridoncosis . . . x 37
Klemmer on . iv 482
Iridoperiphakitis . . . . . x 35
Iris, absence of, case of . . . xvi 320
anatomy of . . . . xi 70
and sclerotic, inflammation of xi 74
coloboma of, appearance of . xvii 235
colour of, in relation to disease v 37
development of, Arnold on . i 551
elasticity of . . . . . x 10
epithelium of . . . . x 12
erectility of . . . . . xv 233
peculiar affection of . . iv 482
excising a portion of . . xi 87, 89
friability of . . . . . v 37
gonorrhœal inflammation of . x 397
in birds, on the structure of . v 553
inflammation of . . xi 70, xx 280
injections of . . . . . xii 532
muscularity of . . . . x 10
ossification of, case of . . xviii 421
physiology of . . . . viii 580
structure and movements of . xxii 284
Irish, crania and aspect of native . xxiv 75
Irish apothecaries' licences, number
of in ten years . . . i 610
dispensaries inefficient . . ii 186
epidemic fevers, history of . xviii 39
hospitals, statistics of . . ii 185
infirmaries, defects of . . ii 186-7
history of . . . ii 186
medical charities, Mr. Phelan on ii 183
corporations, statistics of i 610
Irish medicine, history of . . xviii 37
moss with chocolate . . xiii 87
names of diseases . . . xviii 38
or Carrageen moss . . xiii 65, xvii 21
poor, gloomy picture of . . ii 184
in England . . . xxi 351
sick poor, shocking picture of ii 184
typhus fever, history of . . xviii 38
Iritis, curative powers of mercury in xx 191
cured by bloodletting and mercury ii 348
mercury alone . . ii 348
gouty . . . . . . x 35
mercury in . . . . . x 533
Mr. Lizars on . . . x 539
Mr. Middlemore on . . ii 347
Mr. Tyrrell on . . . xi 70
rheumatic, on tonics in . . xi 74
syphilitic and arthritic, diagnosis of xxiii 418
Mr. Jeaffreson on . . xviii 408
treatment of . . . xx 485
traumatic . . . . . x 35
cysts in . . . . xi 72
Mr. Tyrrell on. . . xi 72
treatment of . . . . xi 71
use of belladonna in . . ii 348
Von Ammon on . . . x 2, 34
his treatment of. . . x 36
Iron, as an element of food . . xvii 4
carbonate of, in hooping-cough vi 222
citrate of, rapid assimilation of xvii 7
how emmenagogue . . viii 250
hydriodate of, very easily decomposed ii 210
hydrated oxide of, an antidote
to arsenic . . . i 594, iv 237
sesquioxide of. . . xiii 74
iodide of . . . . . xiii 73
in albuminuria . . xxiv 44
ioduret of, very easily decomposed ii 210
lactate of, employment of . x 565
muriate of, in softening . . ii 551
oxide of, an antidote for arsenic vii 563,
ix 57, xx 332
presence of in the blood vi 434, 437
protoioduret of, new formulæ for xiii 550
prussiate of, its use in ague . ii 178
red oxide of, for arsenic . . iv 239
rust of, an antidote for arsenic iv 238
saccharated carbonate of . xiii 73
sesquioxide, antidote of arsenic viii 573
sesquioxide of, an antidote x 287, 584
in poisoning . . . x 584
preparation of . . viii 360
value of, in scrofula . . xi 209
Irrationality, stage of, in dementia vii 9
Irregular practitioners, sources of . xix 225
success of . xiv 422, 426
Irreligion of physicians, remarks on xxii 120
Irresponsibility in criminal cases . xix 36
Irrigation, Dr. Macartney's mode of viii 200
in inflammation . . . viii 200
Irrigations, fetid, around Edinburgh xi 1, 19
Irritable breast, remedy for . . vii 530
Irritability an element of disease . xvii 576

Irritability, diagnosis of paralysis by ix 440
Fletcher on the seat of . . v 97
Haller's doctrine of i 387, iii 3, v 490
muscular, Dr. Hall on . . ix 440
M. Longet on . . . xiii 226
remarks on . . . v 505
nervous, from heart and brain xxii 425
of plants, remarks on . . iv 30
organic, definition of . xviii 476
source of, in spinal marrow . ix 440
the brain an exhauster of . ix 440
Irritants, general action of . . xxiv 299
Professor Lanza on . . xxiii 49
Irritated brain, some effects of . iv 62
Irritation, a disorder of the nervous agency ii 3
and excitement, remarks on . iv 429
cerebro-spinal, causes of . xix 101
constitutional, Mr. Travers on ii 1, iii 1
two kinds of . . . ii 3
definition of . . . . xxiii 48
direct, added to reflected . ii 14
and reflected, case of . ii 15
distinction of . ii 3
from chronic change, signs of ii 17
indistinctness of the term . iii 58
in insanity, Dr. Williams on . xxii 55
local, transmission of . . ix 457
Mr. Travers's application of the term ii 4
reflected, definition of . . ii 3
Irritative or pseudo fever . . viii 277
Ischio-cavernosus muscle . . xix 512
Ischl, mineral waters at . . xiv 357
Ischuria, hysterical, in females . xii 122
renalis . . . . . vii 160
cases of . . . . ix 573
singular case of . . ix 551
Isinglass plaster, mode of preparing xiii 265
Mr. Liston's . . xiii 265
Isis revelata . . . . . iii 1
by Mr. Colquhoun . . iii 328
or animal magnetism . iv 441
Islamism and Christianity compared iv 381
Isomorphous bodies . . . xi 142
Isomeric bodies . . . . xi 135, 141
Issue, long, in diseases of the brain ii 596
in the scalp, Dr. Wallis on xvii 96
mode of making xvii 96
Issues, Mr. Liston's mode of forming v 462
Italian Brownism . . . . vii 424
hospitals, theory and practice in xxiv 360
medical institutions . . i 497
journals . . . . i 221, 546
physicians, account of . . iii 576
Italians averse to innovation . . i 498
Italy, climates of . . . . xii 170
Dr. Macculloch's saying of . i 351
mischievous medical theory in ix 226
state of medicine in . . iii 576
statistics of the cholera in . ii 279
ITARD and the savage of Aveyron . xxiv 20
Itch, cured by simple inunction . xxi 530
dissolved caustics in . . iv 515
hydriodate of potash in . . xii 98
nature and treatment of . . iv 513-15
Itch, observations on . . . x 564
sulphur with soap for . . xii 254
the cause of chronic diseases . xxi 232
treatment of . . . . xi 246
at Berlin . . . xii 254
Wismar ointment for . . iv 514
Itch-constitution, remarks on . xx 314
-insect, drugs fatal to . . xii 98
experiments with . . xv 398
Mr. Wilson on . . xv 397
remarks on its discovery . ii 150
Iulus, experiments of Mr. Newport on xvii 185
or galley-worm, structure of . xx 489
Ivory bougies . . . . viii 258
of teeth, development of . viii 166
formation of . . . xx 378
Mr. Nasmyth on . . xii 492
Ivry, private lunatic asylum at . xix 611

JACKSON, Dr. Charles, on inhalation of ether . . . . xxiii 550
JACKSON, Dr. James, his memoir of his son i 209
his moving advice to his son . i 211
on absorption . . . i 156
bloodletting . . . iii 456
typhus fever . . viii 428, 435
JACKSON, Dr. J., jun., a model for students i 212
memoir of . . . . i 209
JACKSON, Dr. J. B. S., on derangement of the spleen . . xxiv 329
JACKSON, Dr. R., his Life and Writings xxi 167
on the economy of armies . xxi 166
JACKSON, Mr. John, his curious analogies xv 457
his erroneous physiology . xv 456
his manifold mistakes . . xv 457
on the spleen, &c. . . . xv 455
problem suggested to . . xv 458
Jacksonian prize for 1835 . . ii 302
for 1837 . . iii 293, vi 590
JACOB, Dr., sent by Q. Elizabeth to Russia i 599
JACOBI, Dr., account of his writings xxii 1
on the forms of insanity . xxii 1
Jacobi, tunica, of the eye, remarks on the ii 352
JACQUEMIN, M., his test of pregnancy iv 68
Jactitation, from undue lactation . xii 329
JADELOT, on diseases of infants . vii 75
JADELOT'S diagnostic traits in children ii 356
Jagellonian University, report from the iii 475
JAGER, Dr., on staphyloma iridis . iv 482
JAHN, Dr., Bacon's prescience of his theory iv 132
basis of his pathology . . iv 122
his classification of diseases . iv 124-6
his new system of pathology . iv 120
his success in practice . . iv 122
mental characteristics of . iv 121
on practical medicine . . viii 419
on the nature of diseases . iv 126-31
opinion on his singular book . iv 130
JAHR'S codex . . . . xxii 567
Jail fever, alleged sources of (App. to vol.) xi 42
Jalap, compound powder of, in dropsy xxi 370
Jamaica medical college . . iii 194
medical statistics of . . viii 213, 216
mortality of troops in . . viii 213

Jamaica, phthisis prevalent in . . viii 220
physical journal . . . iii 193
James, Dr., his Medical Dictionary . i 176
James, Mr., his retrospective address x 463
on inflammation . . . x 463
James, M., on galvanism in paralysis xi 524
Jameson, Mr., on vivesection . xix 248
Jaundice, a mechanical cause of . iii 343
benefit from laxatives in . xi 418
leeches in . . . xi 419
bloodletting in . . . iii 344
causes and prognosis of . . xxi 61
characteristics of . . . xxii 312
classification of causes of . iii 158
coma and sudden death in . xxi 103
diagnosis of, from gall-stones . xxii 317
Dr. Bright on . . . iii 157
Dr. Budd on . . . . xxi 55, 60
Dr. Horaczek on . . . xxii 311
Dr. Stokes on . . . ix 482
duodenitis a cause of . ix 482, xi 418
ending in coma rapidly fatal . i 15
fatal coma in . . . iii 327
from non-elimination . . xii 280
inflammatory, progress of . iii 158
treatment of . . . iii 158
morbid anatomy of . . iii 158
M. Bonet on . . . xii 86
Mr. Twining's discovery in . iii 343
secreting cells of liver in . xv 279
state of the blood in . vi 446, xvii 432
treatment of . . iii 344, xxii 317
the use of mercury in . . iii 344
Jaw, brain-like tumour of . . iv 147
lower, congenital luxation of . xiv 238
old luxation of, reduction of xiv 252
peculiar disease of . . . viii 423
operation for removal of upper xi 196
removal of upper or lower . xi 196
upper, removal of . . . x 284
Jaws, diseases of the . . . xx 195
Jeaffreson, Mr., his multitudinous
cures . . . . . xviii 405
on diseases of the eye . . xviii 405
strictures on his style . . xviii 422
Jealousies and personalities of medical men . . . . . xxi 446
Jeanselme, M., on diseases of the eye x 3, 23
Jefferson College, Philadelphia (U.S.) ii 590
Jefferson, President, on vaccine matter vi 488
Jeffrey, Lord, and grandmamma Wolf ix 199
Jeffreys, Dr., his address . . viii 605
Jeffreys, Mr., his respirator . iii 492
on the statics of the chest xvii 152, 155
Jenner and Bonaparte . . vi 496
and the College of Physicians . vi 496
Jenner, Dr., character of . . xx 349
Baron's account of . . iv 559
early history of . . . vi 478
great injustice done to . . vi 496
his amiable disposition . . vi 496
his bad success in London . vi 481
his bon mot to Mr. Ring . vi 496
Jenner, Dr., his experiments on swine-pox vi 479
final opinion on vaccination vi 497
first case of vaccination . vi 479
first publication on cowpox vi 480
letter to Rev. Mr. Clinch . vi 496
life by Dr. Baron . . vi 477
opponents and supporters vi 487
"Origin of Vaccine Inoculation" vi 488
thoughts on his death . vi 497
honours conferred upon . . vi 488
neglect of the principles of . vi 491
professional thanks, &c., to . vi 490
quotation from his first work . vi 480
remarks on his character . vi 495, 497
on his merits . . . vii 186
votes of parliament to . . vi 489
Jennings, Mr., eulogy on his character iii 168
on buoyancy of the lungs . ii 413
Jersey, Dr. Hooper, on the climate of iv 494
Jews, menstrual punishment of male vi 79
Jobert, Dr., on vesico-vaginal fistulæ ii 561
Jobert's treatment of divided intestine xxiii 21
John, M., his analysis of cerebral matter i 288
Johnson, Capt., the murderer, case of xxiv 236
Johnson, Dr., his Economy of Health v 380, 393
on Bright's disease . . xxiv 321
on hydropathy . . . xxii 428
Johnson, Dr. J., his pilgrimages to
the Spas . . . . xiv 309, 318
on malaria . . . . i 350
strictures on his style . . v 394
Johnstone, Dr., his syllabus of materia medica . . . i 193
on sensation . . . . xi 505
Johnstone on the involuntary motions i 386
Johnstone, Dr., biographical notice of iii 586
Johnstone, Dr. John, death of . iii 586
Joint, external abscess opening into a x 338
formation of an artificial . xx 49
stiff, treatises on . . . xviii 353
Joints, abscesses in, Lisfranc on . xv 49
acute inflammation of . . v 462
and bones, diseases of, treatises on xxi 124
chronic swelling of . . v 462
diseased, actual cautery and
moxa in . . . xxii 69
calomel and opium in . xv 50
compression in . . xv 49
hydropathic baths in . xxii 65
on amputating . . xv 48
on issues near . . v 462
on leeching . . . xv 49
position of . . . xxi 137
treatment of . . v 462, xxii 67
use of rest in . . xv 48
diseases of, causes of . . xxii 63
treatise on . . . xxii 62
in animals . . . xxiv 87
effects of forcibly injecting . xxi 136
immobility on . . xxi 141
excision of articular surfaces of xxii 370
Mr. Syme on . . . iii 482
of smaller . . . iv 230

Joints, experiments on . . . xxi 136, 143
hysterical affections of . . iv 136
inflammation of, iodine in . viii 521
injuries of . . . . . xi 459
chief danger in . . xi 462
and diseases of . . v 461
lacerated wounds of, treatment of xi 460
large, on resection of . . xxi 83
morbid products of, analysis of xxi 126
Mr. Tyrrell on diseases of the iii 146
natural exit of pus from . x 337
new or false, in ununited fractures xxii 166
on opening suppurated . . x 336
on producing anchylosis in . x 336
pathological anatomy of . xxi 125
pus simultaneously in . . x 337
recovered, treatment of . . xv 51
removal of foreign bodies from xi 526
stiff, compression and extension in xviii 363
structure of, Mr. Goodsir on xxii 270
treatises on diseases of . . x 330
Jones, Dr. Bence, on calculi . . xviii 366
on diabetic blood . . . xviii 369
gravel and calculus . . xv 500
oxalate of lime in urine . xx 92
uric acid in urine . . xx 90
Jones, Mr. R., on medical education x 175
Jones, Mr. Wharton, on anatomy of the eye . . . . . x 9
his report on the ovum . . xvi 513
on comparative anatomy . . xii 218
embryology . . . ix 291
impregnation . . . iv 527
inflammation . . . xvii 567
of the eye . . xx 273
ophthalmic medicine . . xxiii 246
staphyloma . . . v 612
the anatomy of the ovum . i 610
the blood . . . . . xiv 585
in inflammation . xviii 255
the eye . . . . . ix 543
the eyeball . . . . x 3
the organ of hearing . . viii 68, 70
the ovum . . . . . ix 2
Jones, Mr. Wm., careless writing of viii 529
on the diseases of women . viii 529
Jones, Professor, his Natural History xix 556
Jörg, Prof., on responsibility of women vii 128
palpable fallacies of . . vii 134
Josse, M., his work on surgery, opinion of iii 130
on cold water in surgery . . iii 114
Journal, American, of Pharmacy . ii 233
French, Military, Medical . xxiii 74
Hebdomadaire, &c. . . . i 547
of Forensic Medicine (German) iii 196
Medicine in general (German) i 546
Medico-chirurgical Sciences i 223
Ophthalmology (German) . ii 232
Practical Medicine (German) i 218
Surgery, &c. (German) . i 218
the Medical Sciences (American) i 228
Quarterly Medical, of India . vi 260
the Indian Medical . . iii 192
the Jamaica Physical . . iii 193
Journal, United States Medical . i 548
Journals, American, reflections on the iii 194
British, list of papers in . . xv 575
medical, list of . . i 223
original papers in . xv 256
colonial and American . . vi 260
medical . . . . ii 234
Danish medical . . . ii 231
foreign medical, account of . i 217, ii 231
French medical . . . i 547
German medical . i 545, ii 232, iii 460
Italian medical . . . i 546
medical, of Spain . . . i 304
on criticism in . . xxii 119
way of augmenting the sale of . xxiv 596
Joy, curative effects of . . . xvi 361
Joy, Dr., on the diseases of arteries xii 114
on diseases of the heart . . xii 112
on spasm of the glottis, referred to ii 240
on the art of prescribing . . xii 120
Judd, Mr., his pathology of gonorrhœa x 385
on syphilis . . . . v 2, 29
Judge, ferocious dictum of a . . v 444
Judges, discrepant opinions of . xix 35
Judgment, false, in physicians . xxii 122
Jugular pulsation . . . . xix 105
vein, case of ulceration of . xviii 367
Jukes, Mr., his remarks on ergot of rye iii 165
on stricture of the rectum . xviii 452
Julie, Mademoiselle, a curer of diseases xxi 534
cases submitted to . . . xxi 539
her system . . . . xxi 535
Julliard, M., on purulent ophthalmia v 31, 38
Jünken on purulent ophthalmia . ii 501
Juries, annoyance-, account of . xv 330
Juries, leet, perambulating . . xv 330
Jurine on pulmonary exhalation . i 242
on watery vapour from the lungs i 242
Jurisprudence, medical, Barzellotti on ix 35, 63
Dr. J. B. Beck on iv 87, viii 246
Dr. Traill on . iii 492
education in . x 196
importance of . xvii 228
indifference to . xvii 229
in England . . ix 54
Mr. Taylor on . ii 422, xvii 228, 232
Mr. Taylor's Manual of xxi 484
term improper . ii 422
Nicolai's manual of . . xvi 68
on the study of . . . xii 174
surgical . . . . ix 61
Jurist, medical, duty of the . . v 172
qualifications of the . x 197
Jurists, medical, recommended . ii 395
Jussieu, his views of mesmerism . vii 321
Juvantia and lædentia, neglect of , xxi 506

Kafirs, form of their skulls . . xviii 386
of Africa, observations on . xxiv 448
Kalmucks, Prof. Pallas's account of . xxiv 460
Kamtschatka yourts, filthiness of (App. to vol.) xi 41

Kangaroo, fœtus of the . . . v 507

KANE, Dr., his Elements of Chemistry xi 514

KANT, his critical philosophy . . v 83

on the mind mastering disease . i 191

Kava, Haonai, victims of . . xviii 210

KAY, Dr., on the blood in asphyxia ii 423

KAYSER, Dr., on the Cæsarean section xiv 199

KEHRER, Dr., on blood-fever . . viii 428, 440

Kelso, health of the poor in . . xiii 576

Kejestein, Lehmann's description of xvii 439

Kelænonesian races of mankind . xxiv 470

Kemaon district in India . . viii 105-6

KENNEDY, Dr., on epidemic cholera xxiii 313

on famine and fever in Ireland xxiv 285

infantile apoplexy . . iii 550

prolapsus uteri . . . viii 596

sounds of the funis . . i 94

scarlatina . . . . xvii 216

KENNEDY, Mr., on diffuse inflammation ix 578

KENNION, Dr., on the optic nerves . vii 282

Kephalosis, Mr. Gardner on . . vii 222

signs of . . . . . vii 223

KERGARADEE, Lejumeau de, notice of i 86

KERST, Dr., his Surgical Miscellanies xv 399

on pathological anatomy . xvi 381, 384

KETTLEBAND, Reg. *v.*, for murder, case of xxi 485

KEY, Mr., his case of deformed tibia . ix 389

his report on syphilitic cases . x 381

on blows on hernial sacs . . xvi 319

bunions and ganglia . . iii 151

excision of elbow-joint . xii 330

lithotomy and lithotrity . iv 350

primary syphilitic cases . xii 335

spinal disease . . . vii 446

ulceration . . . ii 166

Kid, fourteen days old, impregnated xxii 289

KIDD, Dr., his remarks on Galen . ii 603

on the works of Galen . . vi 385

Kidney, Bright's disease of the . v 554

cancer of . . . . . xxii 303

case of deficient, &c. . . xxiv 329

diagnosis of diseases of . . xvi 308

dilatation of pelvis of . . xv 103

diseased, with irritable bladder xiv 162

disease of Dr. Bright, disquisition on ii 225

disease of, diagnosis of . . xx 265

encephaloid matter in . . xi 349

fatty degeneration of, described xxiv 127

interesting case of . . xv 488

M. Rayer on . . . xv 487

inflammatory products in . . xxiv 126

mottling, white degeneration of ii 200

organic disease of, causes of . ii 226

pathological observations on . xxiv 332

structure and changes of . . xxiv 331

structure of, report on . . xxii 267

very common and fatal disease of ii 200

weight and dimensions of . iii 199

wounds of cortical part of . viii 150

Kidneys, action of *infusion* of digitalis on . . . . . xxiii 417

and brain, diseases of . . ix 370

and liver, functions of . . xi 337

Kidneys, and their secretion, report on xix 569

and ureters, singular disease of x 260

anemic discoloration of . . xiii 436

anemotrophous diseased . xi 347

anomalous situation of . . xv 493

atrophy of . . . xv 472, 484

from serous cysts . . ii 461

cadaveric emphysema of . viii 124

calculous deposits in . . xv 472

cancer of, M. Rayer on . . xv 490

cases of diseases of the . . ii 226, 228

total absence of . . xv 492

classification of diseases of . viii 124

congestion of, diuretics in . xvii 495

cysts of the . . . . xv 486

disease of, and diabetes compared ii 227

Dr. Bright's report on . ii 223

in dropsy . . . vii 163

in relation to age . . xi 346

in the fœtus . . . xx 548

treatment of . . . ii 228

varieties of . . . xi 347

diseases of blood-vessels of . xv 486

caution on . . . xi 351

M. Rayer on . . . xv 470

prognosis in . . . xi 350

treatises on . . . viii 121

treatment of . . . xi 350

distinct cellular membrane in . viii 122

sympathies of . . xii 74

Dr. Christison on disease of . viii 121, x 301, 328

enlargement of, in fœtus . xi 227

enormous tumours of . . xv 472

entozoa in the . . . xv 491

experiments on . . . xviii 366

feathery oil-tubes of . . x 221

hæmotrophous diseased . . xi 348

heterologous formations in . xv 488

homologous formations in . xv 487

hypertrophy of the . . . xv 483

hypostatic engorgement of . viii 123

inflammation of . . . x 302

inflammatory diseases of . viii 150

irritable bladder, with disease of xi 360

malformations of . . . xv 492

Malpighian bodies of . . xiv 567

malposition of, case of . . xiv 61

mechanical lesions of . . viii 149

melanosis of the . . . xv 491

mobility of . . . . xv 493

treatment of . . . xv 494

moderate sensibility of . . viii 122

Mr. Corfe's treatise on . . x 221

M. Rayer on diseases of . . x 301, 328

neuralgia of . . . . xv 487

new physiology of . . . x 222

on investigating diseases of . viii 126

organic change in, in dropsy . ii 223

portal system of . . . xiv 567

recovery from wound of . viii 149

secretion by disorganized . viii 123

sign of a wound of . . viii 150

Kidneys, softening of . . . . viii 124
state of, in diabetes mellitus . xxiv 45
weight of . . . . . viii 122
structure and functions of . xx 125
tenderness in region of . viii 122, 126
theological considerations on . x 223
tuberculous disease of . . xv 488
various treatises on diseases of x 301
KIERNAN, Mr., his minute anatomy of the liver . . . iii 292
on the spleen . . . . viii 593
on the structure of the liver . i 395
Kiestein, as a sign of pregnancy xiii 568, xx 525
a test of pregnancy . xii 327, 566
chemical nature of . . xii 327
Dr. Golding Bird on . . xx 69
in urine in pregnancy . . xii 326
KILGOUR, Dr., facetiæ of . . i 357
his advice to mothers . . i 358
his lectures on hygiene . . i 313, 352
on fever in Aberdeen . . xviii 179
on improving a country . . i 353
KILIAN, Dr., his operative midwifery iii 392
his verbosity and pomposity . iii 411
on obstetric exploration . i 85
on tumour of uterus . . ii 571
Kinetic and ideagenic substrata . xix 308
KING, Mr. T. W., his style, notes, and terms . . . . . iv 367
on digestion of the stomach . xvi 310
fracture of thigh-bone . xxiii 419
rupture of œsophagus . xviii 335
strangulated hernia . . vii 456
tricuspid valve of heart . ii 216
valves of the heart . . iv 364
King of Sweden and M. Gama . i 305
King's evil, on the royal touch for xxii 126, 151
KINGSTON, Dr., on pulmonary tubercles iv 151
KINNIS, Dr., his letter on vaccination vii 527
Kino, source of, uncertain . . xvi 358
Kissingen, cheap living at . . xiv 343
mineral springs of . . . xiv 343
wholesome despotism at . . xiv 343
KITTO, Dr., on deafness . . xxi 188
KIWISCH, Dr., on childbed diseases xiii 100, 108
KLEFFENS, Dr. van, on cancer of lungs xv 430
KLENCKE, Dr., on histology . xiv 478, 480
on cod-liver oil . . . xiv 441
on helminthiasis . . . xx 406
on regeneration of tissues . xvi 503
vague idealism of . . . xvi 503
KLEMMER, Dr., on iridoncosis . iv 482
Kleptomania in pregnant women . x 169
KLUGE, Dr., on morbus cœruleus . i 254
on pneumonia of infants . i 253
Knackers of Montfaucon, health of xi 15
Knee, contracted, Louvrier's plan with xiii 14, 16
violent operation on . xiii 19
contractions of, treatment of . xiii 14
extraction of loose bodies from xiv 139
M. Lederle on fungus of . x 330
Knee-joint, amputation at . . xx 55
anatomy of . . . xxii 270
Knee-joint, anchylosed, straightening xx 51
anchylosis of . . . xiii 12
cause of inflammation of . x 334
contractions, causes of . viii 400
cure of . . . viii 400
fatal case of diseased . ii 15
fatality of injuries of . xi 461
gun-shot wound of . . xii 330
loose cartilages in . . vi 171
permanent contraction of iv 169
straightening the . . xxii 68
wounded, case of . . ii 198
Knees, contracted, violent extension of xiii 14-20, 28
KNOLZ, Dr., on cretinism . . xix 372
on the Brunonian system . xix 371
on the cotton-mills of Austria xviii 147
on the plague of Egypt . . xix 366
Knowledge, Bacon's estimate of . ix 429
Dr. Symonds on . . . xxiii 252
love of, Sir H. Davy on . . vi 105
past and present . . . viii 385
pleasure in acquisition of . xxiii 243
use of a retrospect of . . vi 294
KNOX, Dr., on hepatization of the lungs iii 259
on pulsations of the heart . iv 241
KOBELT, Dr., on the organs of copulation xix 507
KÖCHLIN, Dr., on metals as remedies viii 353
KOEHLER, Dr., on incarcerated hernia i 586
KOLK, Van der, on inflammation of eye xviii 48
Konigsberg, university of, account of i 295
KOPP, Dr., on thymic asthma . ii 237
KOPPELIN and KAMPMANN, MM., on arsenic . . . . . xiii 193
Koppian asthma, an improper title . ii 240
KOSTLIN, his microscopic researches xiv 478, 480
Kraft, of Unzer, meaning of the word xxiv 182
'Kram,' sample of, by Professor Kohl i 105
KRAMER, Dr., his mistake on Abercrombie iii 97
his work on the ear, value of . iii 100
on diseases of the ear iii 79, v 216, xxiv 22
Kranken-Haus at Berlin, practice in i 500
KRAUSE, Dr., his anatomical observations vi 218
his Manual of Anatomy iii 198, vi 204
KRAUSS, Dr., on club-foot . . ix 227
Kreasote, different opinions of . i 265
Dr. Elliotson on . . . ii 168
on the topical use of . . vi 235, 270
Kreatic nausea, cause of . . xvii 415
Kreatine, physiological relations of xxiv 265
qualities and properties of . xxiv 265
properties of . . . . xxiv 266
KRIEG, Dr., on amenorrhea . . vii 233
KROHN, Dr. on the iris in birds . v 553
KRONENBERG, Dr., on the nerves . ix 547
KREYSIG, Dr., obituary of . . ix 293
on ischuria renalis . . . ix 551
KUHN, his discoveries in tubercle . iv 318
KUNDT, Dr., on the waters of Ischl xiv 310
Kutkuleja nut in Indian agues . iii 348
KÜTTNER, Dr., on bronchitis of children xii 350
Kyanized timber, danger from use of xi 532
Kysteine, as a sign of pregnancy . xxiii 277

Labia, abscess of, caution on . . xxi 88

and nymphæ, adhesion of . xxi 327

œdema of, in labour, treatment of xix 426

sanguineous tumours of . . xx 532

Labia pudendi, elephantiasis of . xx 548

Labial cancer . . . . xxii 294

glands, diseases of . . . xv 227

Labium, rupture of the, in labour . viii 38

pudendi, fatal wound of . . v 178

Labour, action of abdominal muscles in xix 62

advance of, diagnosis of the . i 110

a peculiar mode of examining in iii 412

aqueous discharge after . . xix 356

artificial, piercing placenta in . x 99

premature . . . iii 415

case of . . . xiv 257

-chairs and cushions . . iii 412

changes of circulation in . xix 423

complicated with enterocele . xxi 87

dangerous practice in . . xii 470

different positions during . xii 471

difficult, a critic's, with a book xxiv 140

deceptive symptoms of . . xiv 369

Dr. Murphy on first stage of . iv 260

enema at commencement of . xix 71

extraordinary, in America . xiii 568

ergot of rye in . . . xii 479

five stages of, described . . xix 63

headache after, treatment of . ii 86

hydrocephalus impeding . xii 480

impeded by tubercles in uterus xii 558

immature, on the term . . vi 82

impediments to . . . xii 480

injury of child's head in . . xiii 548

knots on cord impeding . . xii 480

local bathing after . . . iv 399

management of, report on . xx 528

mean duration of . . . xix 63

mode of inducing premature . iv 404

repressing the head in . iii 407

natural, Dr. Collins on . . ii 74

on the study of . . xii 463

report on . . . xxiii 279

new mode of accelerating . xiv 257

obstructed by vaginal septum . xx 531

on examining per rectum in . iii 412

inducing premature . . iv 411

supporting the perineum in iii 407, xii 470

the hand in abdomen in . iii 407

using instruments in . xii 481

-pains, inefficient, opium in . ii 524

pelvic tumours obstructing . xi 500

premature, Dr. Ashwell on producing ii 199

induction of . . . xiv 366

modes of inducing iii 416, xiii 249

of a dwarf . . . x 272

on the term . . . vi 81

report on . . . xx 534

statistics of . . . xiv 367

-presentations, analysis of . xiii 570

presentation of the head in . xii 464

Labour, preternatural, Dr. Hamilton on iv 405

report on xvii 535-55, xx 529, xxiii 280

prognostic, from vagina of . xii 471

protracted, opium in . . xv 528

strange treatment of viii 471, 473

use of opiates in . . viii 473

purgatives after . . . iv 400

recent literature on . . xvii 535

rigidity in, bleeding for . . iii 407

tartar emetic for . . iii 407

rigidity of membranes in . iv 402

rupture of perineum in . . xiv 369

sad case of neglected . . ii 86

shortness of funis in . . xii 480

size of uterus after . . . iv 466

spirituous lotions after . . iv 399

state of os uteri after . . iv 466

tedious, from obliquity of uterus ii 526

terhoral period of . . . xxiv 115

time of, in Austrian factories . xviii 148

use of the binder in . . iii 407

the roller after . . iv 398

uterine action in . . . xii 478

inefficiency in . . xii 477

variations of pulse during . vii 230

with five children . . . viii 564

imperforate uterus . . iv 375

schirrous uterus . . xi 502

tumours in pelvic cellular tissue . . . xi 504

uterine growths, remarks on ii 200

Labours, causes of death in 164, and table of . . . . ii 74

laborious . . . . iv 400

number of deaths in 16,434 . ii 73

protracted, treatise on . . viii 467

Labourer, effects of his diet on the ii 372, 384

Labouring classes, brevity of life of xv 337

in London, reports on . xi 5

mortality least in . xxi 7

report on health of . xi 1

population, report on the xv 286, 328

Laburnum-bark, case of poisoning by xviii 562

Labyrinth of the ear . . . viii 87

humours of . . viii 79

Labyrinthic cavity of the ear . . viii 78

Labyrinthodon, dental structure in the xx 401

foot-prints of the . . . xx 405

Lac and flores sulphuris, remarks on viii 354

Lacerated or bruised wounds . . xi 310

Laceration of uterus, rare case of . ii 164

LACHESE, Dr., on arsenious acid . viii 568

Lachrymal apparatus, diseases of . xi 76

ducts, obliteration of . . x 29

fistulæ, treatment of . . xxi 310

gland, ducts of . . . xix 586

lower portion of . . x 28

sac, inflammation of, acute . xi 78

chronic . . xi 78

laying open the . . x 29

Lactate of iron, employment of . x 565

Lactation, by mother, obstacles to . x 125

Lactation, case of anomalous . . xii 431

conception during . . xvi 231

effect of, on conception . . xvii 541

fermented liquors in . . xxiv 543

food of mother during . . x 126

influence of catamenia on . xvii 541

mutual benefits of . . . x 125

peculiar sore mouth during . xvii 541

report of M. Dumas on . . xxii 202

report on xvii 274, xx 538, xxiii 291

triennial, remarks on . . xvi 230

undue, amaurosis from . . xii 328

epilepsy from . . . xii 329

headache from . . xii 329

insanity from . . . xii 329

jactitation from . . xii 329

morbid effects of . . xii 328

treatment of . . . xii 329

Lacteal and lymphatic absorption . xix 278

Lacteals, and lymphatics, action of xiv 290

general structure of . . xiv 289

object of . . . . . xxiv 277

Lactic acid, a constituent of flesh . xxiv 268

a specific in gout . . ix 239

diseases from . . . xvi 195

free, in the blood . . xxii 327

from food . . . xvii 33

in relation to gout . . xvi 465

in the stomach . . xxiv 278

a cause of dyspepsia . xi 343

Magendie on . . . ii 208

mode of administering . ii 209

morbid production of . xvi 484

process for procuring . ii 208

properties of . . . ii 208

in relation to lithic acid . xv 506

or acetic acid in stomach . xi 333, 343

Lactiferous tubes of the mammæ . x 116

Lactucarium, remarks on . . iv 106

Lacunæ and canaliculi of bone xxiv 389, 393-5

Lædentia and juvantia, neglect of . xxi 506

Laennec, his discoveries, remarks on ix 399

his discovery and merits . iv 287

his treatise, notes on . . v 423

notes to, by M. Mer. Laennec . iv 298

Laennec, Dr. Mer., on auscultation iv 285

Lafarge, remarks on the case of . xii 176

Lagophthalmus, operation for . xxi 309

La Lancette Française . . . i 226

Lallemand, M., on cystitis . . vi 566

on cure of erectile tumours . ii 564

spermatic discharges . . xv 346

strictures . . . v 572

zoospermata . . . xi 520

Lamarck, M., his theory of species xix 176

Lamb, suckling a, tumours after, case of xxii 157

Lambs, cases of scrofula in . . xxiv 88

Lamprey, respiration of the . . xv 209

Lamps and candles deteriorate air . xix 18

Lancet, rash use of, once prevalent i 398

Lancetted stilettes in stricture . i 534

Land, effects of extreme division of xiv 455

Landowzy, M., on varicocele . viii 253

Land's End, and Worcester, diseases in vi 8

fever in . . . . vi 10, 11

inhabitants of . . . vi 4

medical topography of . vi 3

Lane, Dr., on Materia Medica . ix 541

Lane, Mr., his life at the water-cure xxii 428

Langenbeck, his anatomical plates xiv 539

Langley, Mr., on puncturing smallpox vi 563

Language, and speech, remarks on . vii 228

on due education in . . ix 415

specimen of a new . . . vii 228

Languages of different nations, on the xxiv 52

study of . . . . . xxiv 219

modern . . . x 178

Languor, spasm, &c., Dr. Wilson on xvi 418

Lanza, Dr., his positive nosology . xxiii 37

Lanzani, on the use of cold water . xxii 431

Laplanders, and Monguls, relation of xviii 380

form of their skulls . . xviii 376

Lapps, pyramidal skulls of the . xxiv 75

Lappes, and Finns, different races . xviii 379

Lapis divinus, formula for . . xiii 84

Lardaceous affection of joints . xxi 126, 128

tumours, remarks on . . viii 320

Lardoni, M., his dread of the plague viii 236

Larrey, M., his apparatus for fractures vi 313

his treatment of fractures . vi 312

Larvæ in the bowels, cases of . . xii 219-21

in the human body, origin of . xii 220

of insects, in the human body . xii 219-21

passed by stool . . xiii 540

Laryngeal nerves, Mr. Hilton on the vi 61

sup., effects of section of iii 29

phthisis . . . . . v 432-3

causes of . . . v 433

complication of . . v 434

fatality of . . . xvi 247

four species of . . v 433

observations on . . xviii 293

simple, curable . . v 435

Sir C. Bell on . . . vi 165

symptoms of . . . v 433

pouches of monkeys, use of . xvi 380

respiratory murmurs . . ix 306

ulceration, seats of . . . v 428

Laryngismus stridulus, and convulsions ii 115

authors on . . ii 113

causes of . . ii 116-20

diet in . . . ii 127

Dr. Elsässer on . xvii 377

Dr. Griffin on . v 595

Dr. W. Griffin on . xxi 104

Dr. Ley on . . ii 112, iii 1

facts on . . v 596

glottis in . . ii 114

history of . . ii 114

head affection in . ii 116, 128

in families . . ii 126

iodine in . . ii 127

opposite ideas of . ii 112

pathology of . ii 121

prognosis in . . ii 125

treatment of . ii 125

Laryngitis, acute, bronchotomy in . v 430
diagnostic table of . xviii 288
symptoms of . v 430, xviii 287
treatment of . v 430, xviii 288
use of caustic in . . xxiv 502
cases of, tracheotomy in . . xx 91
chronic, account of . . v 432
Dr. Roots on . . . iii 150
error on . . . . xviii 293
insufflation in . . . v 436
lesions from . . . v 432
lunar caustic in . . v 435
mercury in . . . v 436
stethoscopic signs of . v 434
symptoms of . . . xviii 292
treatise on . . . v 424
treatment of v 435, xvi 247, xviii 295
whispering in . . . v 435
cupri sulphas valuable in . . i 569
divisions of . . . . xviii 287
Dr. Chavasse's case of . . xviii 290
Dr. Graves on . . . xvi 247
erysipelatous . . . . v 431
fatal case of . . . . xxii 468
follicular, symptoms of . . xxiv 496
in the aged, fatality of . . xvi 247
œdematous . . . . v 431
diagnosis of . . . xviii 296
scarification in . . xviii 298
treatment of . . v 431, xviii 297
on early tracheotomy in . . xviii 291
operations in, by Dr. Wilson . xv 151
scarifications in . . . vii 551
stridulous, discrepancy on . xviii 299
M. Valleix on . . . xviii 298
Laryngotomy, for scalded glottis . vi 270
history of . . . . xvi 51
observations on . . . v 441
operations of . . . . xii 260
two cases requiring . . xvi 326
Larynx, acute affections of, spasm in v 431
and fauces, tubercles in . . xi 239
and pharynx, anatomy of . . xxiv 483
and throat, on diseases of . xxiv 482
and trachea, Mr. Porter on . v 423, 427
Mr. Ryland on . . v 424, 428
works on . . . iv 285
on wounds of . . v 465
a new muscle of the . . xxii 270
cancerous ulceration of . xviii 294
case of closure of . . . xiii 243
fistula of . . . xiii 243
pebble in . . . iii 265
closure of, speech in . . xiii 243
croton oil in affections of . ii 554
disease of, Sir C. Bell's case of xxiv 498
diseases of, M. Trousseau, &c. on v 424, 428
erysipelas of, bronchotomy in . v 432
treatment of . v 432
external fistulæ of . . . xiv 232
follicles of, ulceration of . . xxiv 496-7
foreign bodies in, diagnosis of . xi 452
functions of the nerves of . xiii 226
Larynx, mode of applying caustic to xviii 296
necrosis of cartilages of . . v 432
syphilitic ulceration of . . xviii 295
ulcers and erosions of . . v 429
ulcers in, &c. . . . xxiii 431
varieties of glands in . . xxiv 123
LASSAIGNE, M., on detecting arsenic xiii 191
Latex, of plants, circulation of the . iv 27
vessels of the, in plants . . ix 504
LATHAM, Dr., on clinical medicine . ii 473
on diseases of the heart . xx 175, xxiii 169
specimen of his lecturing . ii 478
Lactiferous tissue, Dr. Carpenter on xiii 163
Latin examinations, remarks on . ix 418
poem, recited at Gottenburg . x 550
Latin, and Apothecaries' Company . i 519
doggish, in medical rescripts . xxiv 506
medical student's guide to . i 519
on the study of, by apothecaries ii 607
Latitudes, mean mortality in . . xiv 448
Laudanum, poisoning a child by, case of xxiii 299
by minute doses of . xiii 574
sale of, in Manchester . . xxi 355
LAUER, Dr., on the head of thigh-bone iv 205
Laughter, consensual nature of . xxii 515
LAURA BRIDGMAN, account of . x 288
Laurel water, account of . . xiii 59
LAURIE, Dr., on homœopathic medicine xxi 225
Lauro-cerasus, use of, in neuralgia . i 263
LAUTERBOURGS, the, magnetisers . vii 312
LAVACHERIE, M., on white swellings xii 512
LAVOISIER, on pulmonary vapour . i 242
on watery vapour from the lungs i 242
LAW, Dr., on mercury . . . vii 581
LAW, J. T., his address at Birmingham i 203, 206
LAW, Mr., on arsenic in plethora . v 605
Law, true signification of the word . v 101
where the term objectionable . v 357
Laws, empirical, deduction of . . xx 142
of nature, Mr. Carpenter on . vii 172
observations on . . xix 159
LAWRENCE, Mr., on ruptures v 547, xiv 91, 110
on venereal diseases of eye . x 381
LAYARD, Dr., on variola in animals vi 482
Laybach, diseases at, in 1828 . . x 204
LAYCOCK, Dr., his analogies . . xii 66
objections to doctrines of . xii 68
on colchicum . . . viii 280
function of the brain . . xix 298
hysteria . . . . v 601
natural history of disease . xxi 525
nervous diseases . . xii 60, 77
proleptics . . . . xviii 165
saliva . . . . v 268
style of . . . . . xii 61
Lazereth, new, of St. Petersburgh . xxi 466
Lead, acetate of, and opium, in pneumonia xx 81
detected in blood . v 59
in cholera . . v 280
in fever . . . v 239
its use in bronchitis . x 288
its use in hernia . vi 537
large doses of . . x 430

Lead, acetate of, poisoning by . . xii 249
variable effects of . v 58
action of acetate of . . iv 208
water on . . vii 448, xviii 546
anæsthesia from . . . x 453
carbonate of, on poisoning by . xviii 546
cerebral affections from . . x 454
colica pictonum from . . xviii 549
coloration of gums by . . x 427
delirium from . . . x 455
detected in milk of a cow . xii 348
detection in the fluids, tissues, &c. x 444
of, in the brain . . x 460
diseases occasioned by . . x 426
effects of, on animals . . v 59
on the system . . ix 435
internal use of preparations of . x 430
introduction of into the system v 57
iodide of, pills of . . . xiii 364
its effect on the gums . . xi 450
its introduction into the system x 430
ligatures of, in palate fissures xi 196
marks of system affected by . x 427
naturally in the body . . xi 226
new treatment of diseases from ii 576
on non-absorption of by the skin x 429
preparations of, causing colic . x 428
remedial use of, caution in . x 430
report on poisoning by . . xviii 546
sugar of, a bad collyrium . xvii 234
workmen in, on the colic of . ii 573
Lead-colic, complications of . . x 440
contraction of intestines in v 61
description of . . . x 427
diagnosis of . . . x 441
Dr. Grisolle on . . v 55
enema for . . . v 64
from ingestion of aliments x 430
inhalation . . x 431
minium and cerusse . v 57
historical sketch of . . x 428
in France, statistics of . ii 573
in relation to age . . v 57
lesions of innervation in . v 60
means of preventing . v 62
mode of its production . x 428
nature of . . . v 61
not generally fatal . . ii 573
occupations subject to . x 431
post-mortem appearances in x 443
precursory signs of . . ii 575
predisposing causes of . x 435
preservatives against . ii 575
prevention of, in manufactories x 433
prognosis in . . . x 442
progress and duration of . x 440
proportion of deaths from v 62
queries and answers on . ii 573-75
relapses of . . . x 441
frequent . . ii 573
relief in, from pressure . v 59
remarks on . . . xvii 416
retraction of abdomen in x 438
Lead-colic, seat and nature of . . x 444
state of the tongue in . v 59
sulphuretted hydrogen in ii 575-6
sulphuric acid in . . x 445
not preventive of v 63
symptoms of . . . x 437-40
Tanquerel's formulæ for . x 446
the pulse in . . . v 60
treatment of . . . v 64, x 445
in La Charité x 446
unaffected by season . v 56
vomiting in . . . v 60
Lead-paralysis, account of . . x 449-51
lesions in . . . x 452
prognosis in . . . x 452
progress of . . . x 451
treatment of . . . x 452
varieties of . . . x 451
Lead-poisoning, amaurosis from . x 454
catalepsy from . . x 457
coma from . . . x 456
convulsions from . . x 457
epilepsy from . . . x 457
Lead-rheumatism, description of . x 448
diagnosis of . . . x 448
or neuralgia . . . x 447
treatment of . . . x 449
Leaden pipes, on water supplied through . . . . xviii 546
Leamington, waters of . . . xiv 345
Leanness from short intestinal canal xi 355
Learning, the three distempers of . iv 132
Leaves and leaf-stalks as food . xvii 20
Lebaudy, Dr., his anatomy of the regions, &c. . . . . ii 196
Lebert, Dr., his pathological physiology . . . . xxii 18
Le Canu, M., on the blood . . vi 431
Lecturer, the honest, and pretender ii 481
Lecturers, medical, fittest age of . x 194
number of in Prussia i 296
Lectures, American . . . iv 194
clinical, Dr. Latham's . . xx 175
curtain, salubrious . . . i 357
Dr. Armstrong's . . . i 34
in the United States . . ii 594
introductory, at medical schools i 203
Lederle, M., on fungus of the knee x 330
Ledermuller, his discoveries . xiv 485
Leech-bites, hemorrhage from, stopping xxiii 375
Leech, club-shaped bodies in ganglions of vi 413
medicinal, dental apparatus of xxiv 440
the German, described . . vi 594
the horse, anatomy of . . xxiv 440
the Hungarian described . vi 594
Leeches, application of . . . xiv 3
to os uteri . iii 428
danger of using old . . x 563
divided, experiments on . . xii 527
not reproduced . . xii 527
eight years to full growth . i 282
enormous application of . . vi 516
how brought to Paris from Pesth i 282

Leeches, import and export of, in France iii 448
in the nose, headache cured by iv 251
on increasing them in Paris . i 281
poisoning of by morbid blood xii 247
preference of, by Lisfranc . i 282
Prussian regulations on . . vi 593
to cervix uteri, Duparcque on ii 516
how applied . ii 516
to fractures, on applying . xiv 4, 9
to lining of nostrils, use of . ii 173
to os uteri, objections to . xxiv 153
to tumours, remarks on . . xiv 4
used, mortality of . . . xiii 234
use of, in gonorrhœa . . ix 240
in syphilitic ulcers . . ix 240
weight of . . . . . vi 594
whence supplied to France . i 281
within rectum, mode of applying xxiv 512
Lee, Dr., his clinical midwifery . xv 226
his elements of geology . . xi 224
error on the placenta . vi 83
lectures on midwifery . xvii 527
physiology for schools . viii 533
on abortion . . . . . vi 81
diseases of the uterus . x 249
fibro-calcareous tumours of uterus ii 165
polypus of the uterus . ii 165
puerperal ophthalmia . xxiii 413
supernumerary mammæ . v 594
the corpus luteum . . ix 445
the nerves of the uterus . xi 493
Lee, Mr. A., his translation of Celsus iii 204
Lee, Mr. Edwin, his book recommended to students . . i 500
his memoranda on France, &c. xiv 309
his translation of Martin . . xiv 527
on mineral waters . . . iii 202
stammering and squinting . xii 209
the continental institutions i 494
the mineral springs of England xiv 309
Lee, Mr. T. S., on tumours of the uterus xxiv 506
Leeds, Philosophical Society of . v 211
Leeson, Mr., on Liebig's physiology, &c. xxii 221
Leet perambulating juries . . xv 330
Leeward islands, health of troops in viii 213
Le Fay's pomatum, notice of . vi 500
Lefevre, Dr., his history of medicine in Russia . . . i 597
on medicine in Russia . . iv 263
Lefevre, Sir George, his apology for the nerves . . . xix 240
his thermal comfort . . xv 541
Leg, amputation of, place for . xiii 565
atrophy of, case of . . xvi 267
deformity of, cured . . ix 389
new artificial . . . . xii 541
separation of at knee-joint, case of iv 257
swelled, observation on . . xxi 92
Legs, amputation of both . . vi 550
Legal medicine, causes of ignorance of xii 174
Devergie's definition of . ii 392
in France and England . xii 174
treatises on . . . ii 390
Legal medicine, various definitions of ii 391
or juridical medicine, remarks on x 196
Legallois, Dr. Philip on his experiments ii 131
his experiments on nerves . ii 131
on the principle of life . . v 530
on the spinal marrow . . v 530
Legitimacy, jurisprudence of . . ix 42
Legumes, nutritious properties of . xvi 283
Legumin, composition of . . xvi 283
in the almond . . . xvi 283
nutritive properties of . . xvi 283
Leguminous seeds, constituents of . xvii 19
L'Heritier, M., a trait of presumption in . . . . . xvii 439
on pathological chemistry xvii 424, 425
Lehmann, Dr., on pathological chemistry . . . xvii 424, 426
Lehmann, M. von, on treatment of lunatics . . . xi 104, 125
Lehrs and Scharlau on Schönlein xxi 20
Leicester, cold-water cure of fever at xxiii 269
Leighton, Mr., on the medical sciences vii 525
Leipzig annals of medical science . iii 462
Leitao, Dr., on intermittent fevers v 558
on the influenza in Lisbon . v 556
Lemon-juice in pruritus scroti . xii 548
Lendrick, Dr., on the nitro-muriatic bath . . . . . iv 254
Lens, crystalline, anatomy of the . vi 207
development of . . vi 209
dislocation of . . ii 350
effect of loss of . . xxiv 368
minute structure of . ix 511
polarizing structure in . ii 599
structure of . . . ii 218
dislocation of the, case of . xiv 252
fibrous structure of . . vi 208, 210
inflammation of, Mr. Tyrrell on xi 79
membranous part of the . . vi 207
ossification and dislocation of . xxiii 425
reproduction of the . . viii 247
structure of the . . . xxii 286
Lenses, kinds of, for defective sight xvi 362
pebble or Brazilian-quartz . xvi 362
Lenticular capsule, wounds of the . v 36
Leo, Dr., his unhappy fate in Russia i 598
Lepelletier, M., on emetic tartar i 415
Lepra, and psoriasis, Mr. Wilson on xv 395
treatment of . . . xv 395
effect of civilization on . . xviii 254
forms of, in Russia . . iv 227
in Bergen, Dr. Danielson on . xviii 346
Mr. Plumbe's ointment for . xv 396
syphilitic . . . . xxiii 361
tincture of cantharides in . xv 396
use of arsenic in . . . xv 396
Lepra albaras ulcerosa, case of . iv 228
Arabum tuberculosa, case of . xxiv 48
crustosa, case of . . . iv 229
elephantiasis leonina . . iv 228
localis pedum, case of . . iv 227
tuberculata virulenta . . xii 424
ulcerosa, case of . . . iv 227

Leprosy and leper hospitals . . xiii 563

British . xiii 256

antiquarian notices of . . xiv 244

at Patna, in India . . . xix 73

deaths in England from . . xix 356

in royal families . . . xiv 244

Norwegian, cause of . . xviii 351

description of . xviii 347, 349

Dr. Boeck on . . . xviii 346

hereditary . . . xviii 351

treatise on . . . xiii 461

treatment of . . . xviii 352

originating from yaws . . ix 531

Leroy d'Etiolles, M., his inventions in lithotrity . xii 408, 411

his lithoprione . . . xii 411

his unfairness to Ducamp . xii 409

on calculus . . . xii 388, 394

on urology . . . . xxii 233

Lesions, Prof. Lanza on . . xxiii 56

vascular, law of symmetry of . vi 49

L'esthiomene, treatment of . . xv 36

Lestocq, Dr., his tragical fate in Russia i 604

Letter from Heidelberg, extract of . iv 282

Letters to a Mother on the Care of Children . . . . . ii 221

Levator pharyngis internus muscle . xiv 376

Lever-belt for spinal deformity . xvi 347, 350

Lever, Dr., on diseases of the uterus xvii 506, 513

on pelvic inflammation . . xviii 341

on pelvic tumours . . . xviii 333

on protracted labour . . xxiii 424

on puerperal convulsions . xviii 340

Lever, Mr., his obstetrical statistics xii 343

on pelvic encysted tumours . xi 463

on pelvic tumours . . . xvi 310

on uterine hemorrhage . . xvi 320

Lever, Mr. King's, for the pulse . v 333

Levin, M., on transmissible diseases xiii 28

Leucoma treated by incisions . xiv 558

Lévy, Dr., on hygiene, public and private xix 51

Leucorrhea, an astringent wash for x 45

a variety of . . . . xix 347

bloodletting in . . . viii 41

Dr. Ashwell on . . . xix 346

Dr. Burne on . . . x 44

Dr. Meigs on . . . viii 41

history of . . . . ii 553

infantile, case of . . . vi 87

described . . . vi 87

male infection by . . . xi 181

observations on . . . xiv 388

pain of left side in . . . x 44

relaxation from . . . vi 88

report on . . . . xxiii 293

rheumatism from suppressed . xviii 522

root of the elder in . . iii 508

simple, Dr. Locock on . . vi 88

Sir C. Clarke on . . vi 88

spinal irritation from . . xii 106

the discharge in . . . v 89

treatment of . . . iii 401, iv 369

Leeuwenhoeck, his discoveries xiv 482, 488

Leupoldt, Dr., his characteristics of medicine . . . xxiii 86
Leuret, M., his lunatic asylum . xix 610
Lewins, Dr., his letter on colchicum iv 565
on colchicum . . . iv 249
Lexicon of living medical authors . xvii 504
Ley, Dr., on laryngismus stridulus ii 112, iii 1
death of . . . . . iii 586
Leyden, University of . . . vi 275
Liberty, civil, favorable to health . i 361
of the subject, remarks on . xix 229
Library for medical practitioners, Danish i 227
of medicine by Dr. Tweedie x 231, xii 92-120
literary faults of . . xii 108, 120
of Practical Medicine, German i 218
of the College of Surgeons . iv 191
the American medical . . iv 495
Lice, remarkable formation of . xii 432
License, &c., examinations for the . xix 549
Licensed practitioners, registration of xix 550
Lichen and strophulus, M. Rayer on ii 154
species of . . . xv 391, 392
syphilitic . . . . . xxiii 360
Lichens, alimentary properties of . xvii 20
'L'idée mère' defective in medicine vi 294
Liebig, Prof., his Animal Chemistry xiv 492, xxiii 492
his Chemistry of Food . . xxiv 264
doctrine on motion denied xiv 498
Letters on Chemistry . xvii 224
Organic Chemistry . . xi 436
physiology travestied . xxii 221
prescription for soup . xxiv 272
Turner's Chemistry . . xxiii 584
unjust sneer, on endosmose, &c. xxiii 385
meed of praise to . . . xiv 493
Mulder on . . . . . xxiv 273
objections to his doctrines . xv 503
on animal chemistry, fault of . xiv 493
on the food of animals . . xxiii 157
physiological flight of . . xiv 493
Lieberkuhn's glands in intestines . i 526
Lieberkuhn, his discoveries . xiv 485
Life, active, its effects on longevity i 198
admeasurement of . . . iii 42
Andral's error in . . iii 51
best in England . . iii 42
errors common in . . iii 51
Louis' errors in . . iii 51
agents supporting . . . iii 48
and action, Prof. Muller on . v 89
and vitality, remarks on . . xiii 161
animal and vegetable, Dr. Shearman on . . . . . xxi 473
animal, organs of, report on . xvii 261
at the water cure, by Mr. Lane xxii 428
caloric in relation to . . xviii 518
capacity of organizing . . xxi 545
death, and dormant vitality . xv 137
duration of, subject to fixed laws i 203
Dr. Arnold on . . . v 90
Dr. Campbell on the cause of xiv 182
Dr. Fletcher's idea of . . v 94

